# Integrating Vision‐Language Models for Accelerated High‐Throughput Nutrition Screening

**DOI:** 10.1002/advs.202403578

**Published:** 2024-07-08

**Authors:** Peihua Ma, Yixin Wu, Ning Yu, Xiaoxue Jia, Yiyang He, Yang Zhang, Michael Backes, Qin Wang, Cheng‐I Wei

**Affiliations:** ^1^ Department of Nutrition and Food Science, College of Agriculture and Natural Resources University of Maryland College Park MD 20742 USA; ^2^ CISPA Helmholtz Center for Information Security 66123 Saarbrucken Germany; ^3^ Netflix Eyeline Studios Los Angeles CA 90028 USA

**Keywords:** food analysis, high‐throughput screening, machine learning, precision nutrition, vision‐language model

## Abstract

Addressing the critical need for swift and precise nutritional profiling in healthcare and in food industry, this study pioneers the integration of vision‐language models (VLMs) with chemical analysis techniques. A cutting‐edge VLM is unveiled, utilizing the expansive UMDFood‐90k database, to significantly improve the speed and accuracy of nutrient estimation processes. Demonstrating a macro‐AUCROC of 0.921 for lipid quantification, the model exhibits less than 10% variance compared to traditional chemical analyses for over 82% of the analyzed food items. This innovative approach not only accelerates nutritional screening by 36.9% when tested amongst students but also sets a new benchmark in the precision of nutritional data compilation. This research marks a substantial leap forward in food science, employing a blend of advanced computational models and chemical validation to offer a rapid, high‐throughput solution for nutritional analysis.

## Graphical abstract

This study integrates vision‐language models with chemical analysis techniques to revolutionize nutritional profiling. Utilizing the extensive UMDFood‐90k database, the novel UMDFood‐VL model significantly enhances the accuracy and speed of nutrient estimation, reducing experiment time by 36.9% and achieving a macro‐AUCROC of 0.921 for lipid quantification. This approach sets a new standard in precision nutrition and food science.

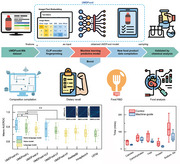

## 1 Introduction

The pursuit of accurate nutritional information underpins effective dietary guidance and public health policy.^[^ ^1^ ^]^ However, the integrity of current food composition databases is undermined by significant limitations: they predominantly feature basic nutrient facts and omit critical data on functional nutrients.^[^ ^2^ ^]^ This shortfall is further exacerbated by the introduction of over 100000 new food products annually, each requiring comprehensive chemical analysis—a task that collectively exceeds $100 million in global expenditure.^[^ ^3^ ^]^ Direct analysis, despite its accuracy, is resource‐intensive and inefficient, burdened by high costs and the necessity for skilled analysts to conduct precise experiments.^[^ ^4^, ^5^ ^]^ Conversely, indirect analysis, which relies on literature and laboratory reports, often fails to accurately capture the unique nutrient compositions of new food products, thereby falling short of the rigorous standards required for reliable nutritional information.^[^ ^6^, ^7^ ^]^ Thus, accurate chemical analysis platforms are extremely important for high‐throughput screening of food products.^[^ ^8^ ^]^

Recent advancements in machine learning, particularly with the advent of sophisticated transformer‐based models like generative pre‐trained transformer (GPT),^[^ ^9^ ^]^ self‐supervision language‐image pre‐training (SLIP),^[^ ^10^ ^]^ bootstrapping language‐image pre‐training (BLIP),^[^ ^11^ ^]^ large‐scale image and noisy‐text embedding (ALIGN),^[^ ^12^ ^]^ object‐semantics aligned pre‐training (OSCAR),^[^ ^13^ ^]^ and contrastive language‐image pre‐training (CLIP),^[^ ^14^ ^]^ present a promising shift towards automating and refining the process of food data compilation. These technologies offer a path to significantly reduce the labor and error associated with traditional methods.^[^ ^15^, ^16^, ^17^ ^]^ Nonetheless, the deployment of these models for nutrition estimation and dietary recall is in its infancy, with several professional and technical challenges impeding their widespread application.^[^ ^18^ ^]^ These include the potential for misinformation, difficulties in interpreting model outcomes, and the need for substantial retraining of personnel, highlighting a pivotal gap in leveraging these technologies for effective nutrition data analysis.^[^ ^16^, ^19^ ^]^

**Figure 1:**
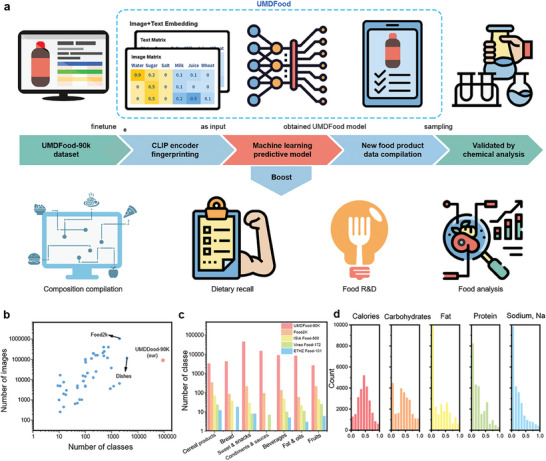
Research Framework and UMDFood‐VL Model Overview. a) This diagram delineates the research pipeline, highlighting the UMDFood‐90k dataset collection and the development of UMDFood models to enhance composition analysis. These advancements support applications in precision nutrition, food research and development (R&D), and analytical food science. b) UMDFood‐90k dataset in relation to existing food databases, showcasing a tenfold increase in diversity, as reflected in the number of unique food product classes.^[^ ^20^, ^21^ ^]^ c) Categorical comparison between UMDFood‐90k and conventional food databases, demonstrating the enhanced variety in UMDFood‐90k. d) Normalized nutrient distribution within UMDFood‐90k, offering a quantitative visualization across various nutrient types.

Addressing these critical challenges, this study introduces UMDFood‐VL, a novel vision‐language model (VLM) designed to streamline food chemical analysis by utilizing front‐of‐package labeling and product images for the estimation of 11 essential nutrients (**Figure** **1**a). Built on the UMDFood‐90k database, the largest multimodal food database to date, UMDFood‐VL achieves remarkable accuracy, as evidenced by a macro‐AUCROC of up to 0.921 in fat estimation. Moreover, it ensures nutritional estimates deviate less than 10% from chemically analyzed values for more than 82.2% of the products assessed. Significantly, UMDFood‐VL facilitates a 36.9% reduction in experiment time, underscoring its potential to drastically improve the efficiency and cost‐effectiveness of nutritional data compilation. This innovative approach not only meets the urgent need for updated and precise food composition databases but also marks a substantial leap forward in nutrition science, offering a scalable solution to the challenges introduced by the rapidly growing food product market.

## 2 Results

### 2.1 UMDFood‐VL Outperforms the State‐Of‐The‐Art Method of Food Compilation

We propose that an effective integration of visual and compositional data is essential for the interpretation of distinctive attributes of food products. To demonstrate this theory, we undertook the UMDFood‐90k dataset, an expansive collection comprising 89533 food products. This dataset, capturing more than 99.9% of the branded food product spectrum in the United States, covers 35 major categories and 238 specific types, as defined by the USDA Branded Food Database (Figure 1b–d). It significantly surpasses existing datasets in both the diversity of food product categories and the volume of items within each category, providing a robust basis for further model development. On average, each product in this dataset contains 12.8 ingredients, with the 100 most frequent ingredients displaying a remarkably low document frequency of just 12% (Figures S1,S2, Supporting Information). Such considerable ingredient sparsity poses a complex challenge to traditional deep‐learning models for nutrient estimation.

**Figure 2:**
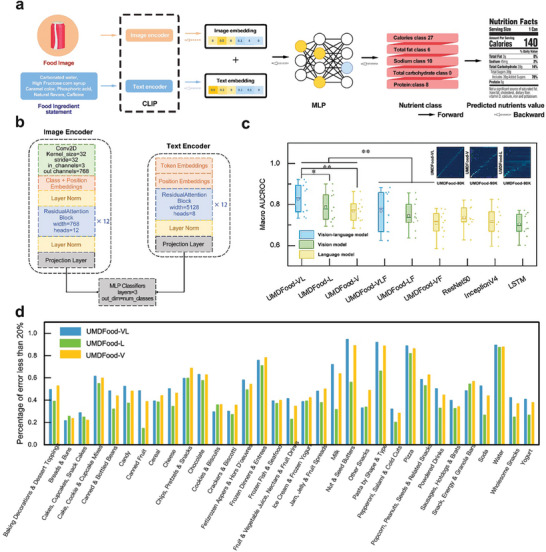
Schematic Representation of the UMDFood‐VL Model and Comparison Analysis. a) The UMDFood‐VL framework integrates a CLIP model with a multilayer perceptron (MLP) classifier.^[^ ^13^ ^]^ Using food product images and ingredient statements, the CLIP model produces corresponding embeddings that serve as inputs to a 3‐layer MLP classifier. The CLIP model, initially pre‐trained with weights from OpenAI's CLIP, is further refined in conjunction with the MLP classifier through cross‐entropy loss on the UMDFood‐90k dataset. b) Details of the UMDFood‐VL model architecture, depicting both image and text encoder structures. c) Comparison analysis of various model performances in nutrient estimation tasks using the UMDFood‐90k dataset. The primary graph displays macro‐AUCROC statistics across different models for 11 nutrient estimation tasks, while the inset shows the error matrix for calorie estimation using UMDFood‐VL, UMDFood‐V, and UMDFood‐L, taking UMDFood‐90k as the reference. d) The percentage of items within each category where the UMDFood‐VL model‐estimated values deviate less than 10% from UMDFood‐90k values. Additional error matrices for all nutrients and models are available in the Supplementary Materials.

Following the dataset compilation, we introduced the UMDFood‐VL, an advanced VLM, designed to accurately estimate nutrient values (**Figure** **2**a,b). The efficacy of this model was evaluated using the macro‐AUCROC metric (equal weight to each nutrient class), wherein UMDFood‐VL exhibited a significant improvement over baseline models, achieving significance testing *p* < 0.08 when compared to UMDFood‐L (only text data as input), and *p* < 0.05 in comparison to all other models. It achieved an average nutrient estimation performance macro‐AUCROC score of 0.827, with fat estimation accuracy being exceptionally high at 0.921. However, its performance in calorie estimation was relatively modest, marked at 0.709. A comparison of the error matrices for UMDFood‐V (only image data as input) and UMDFood‐L models revealed issues related to hierarchy and classification scheme imbalances in the data, despite adjustments made during training to account for these imbalances. Accurate estimations are indicated by a concentration of high percentages along the error matrix diagonal. At the categorical level, UMDFood‐VL outperformed the other model variants in 21 out of 35 categories, with 4 categories showing more than 80% accuracy with less than a 10% discrepancy between the estimated values by UMDFood‐VL and the ground truth values annotated in UMDFood‐90K.

### 2.2 Interpretive Analysis Reveals the Relationship Between Input and Output Nutrient Values

**Figure 3:**
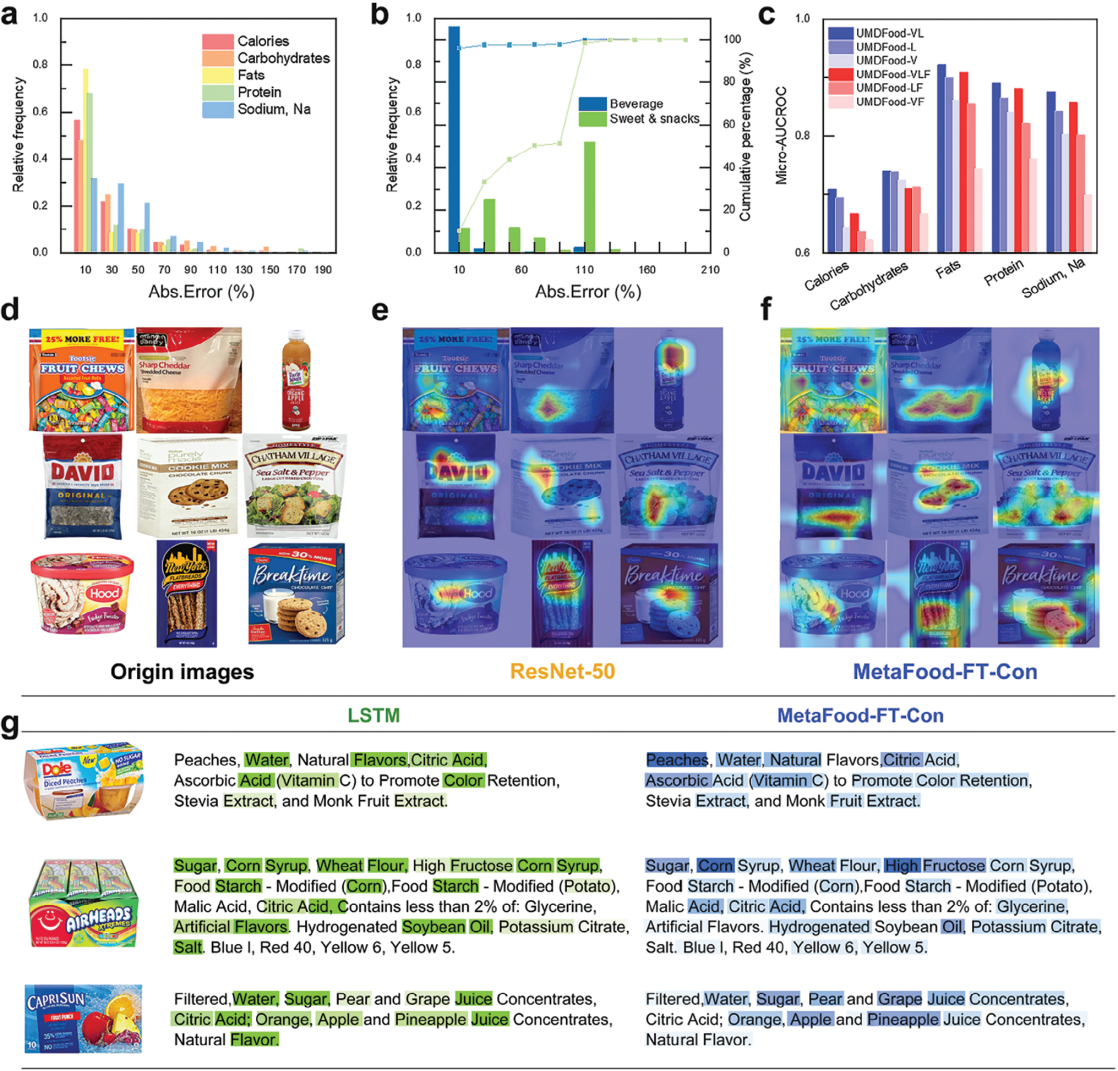
Interpretative Analysis of UMDFood Models Using Error Distribution and Attention Visualization. a) The absolute error distribution for nutrient estimations conducted by the UMDFood‐VL model indicates the variability and precision of the model's nutrient predictions. b) The absolute error distribution in the estimation of total carbohydrate values within the beverage and sweet & snack categories from the UMDFood‐90k database highlights the superior performance of UMDFood‐VL in beverage products. c) Comparison of the micro‐AUCROC scores of various models for the estimation of selected nutrients (n = 5), showcasing the model's efficacy across different nutrient types. d–f) GradCAM visualizations of model attention across selected product images, highlighting the focus areas of ResNet‐50 in UMDFood‐VL, thereby revealing the nuanced interpretative capabilities of the latter. g) Comparison of the ingredient texts weighted by LSTM in UMDFood‐VL models for several food products, demonstrating the distinct emphasis each model places on different ingredients in their encoding and reasoning.

In order for experts in food analysis to intuitively understand the effects of the model and to meet the needs of regulations in practice, the model is interpreted at different levels of analysis.^[^ ^15^ ^]^ First, we detailed the frequency distribution of absolute errors generated by the model. In the domain of fat value estimation, the model exhibited remarkable precision with 78.0% and 86.6% of items in UMDFood‐90k displaying absolute errors of less than 10% and 30%, respectively, thus meeting the stringent accuracy requirements necessary for subsequent chemical analysis (**Figure** **3**a). Further investigated UMDFood‐VL's performance across various food categories. The model showed notably higher precision in estimating carbohydrate values in beverage products, with 96.0% of these products having an absolute error margin under 10%. Conversely, only 10.4% of sweet and snack products met this criterion, indicating a significant variance in model accuracy across different food categories and suggesting model refinement and optimization (Figure 3b). These findings indicate a significant variation in the model's precision across different food categories, suggesting potential areas for further refinement and optimization (See comprehensive comparison of model performance in Supporting Materials).

We also explored the impact of fine‐tuning on the efficacy of the CLIP model. Initially employing the pre‐trained CLIP model as a mere feature extractor, we noted its configuration in three variations: UMDFood‐VLF, UMDFood‐LF, and UMDFood‐VF, where it served to capture embeddings from food images and ingredient lists for a Multilayer Perceptron (MLP) classifier predicting nutrient values. Employing micro‐AUCROC metrics for a granular analysis revealed that the absence of fine‐tuning significantly impaired the model's performance across all nutrient estimations. In contrast, calorie value estimation benefited most from fine‐tuning, indicating the indispensability of this process for enhancing predictive accuracy in nutrient value estimation (Figure 3c).

To better illustrate the model's nutrient estimation approach using product images, we integrated GradCAM to produce coarse localization maps. These maps effectively highlight the model's “attention regions” within a food image. A notable observation was that the inclusion of ingredient text information significantly altered the model's focus areas (Figure 3d–f). In comparison with conventional convolutional neural networks (CNNs), UMDFood‐VL displayed more comprehensive attention to key food features presented on the package, such as specifically highlighting the cheese and cookie areas in the provided example images. In contrast, the attention mechanism of CNN models tended to be overly concentrated, often zeroing in on a singular feature within the image, such as croutons in a salad or the brand logo in a nut product image, thereby demonstrating a lower accuracy.

Lastly, an analysis of ingredient weight allocations in UMDFood‐VL, compared to a traditional LSTM model, revealed a more nuanced extraction of food ingredient features by UMDFood‐VL. This was visualized through a color‐coded transparency method, indicating the weights assigned to each ingredient (Figure 3g). UMDFood‐VL's approach to ingredient weighting highlighted its ability to discern more specific and distinctive ingredients, thereby enhancing the model's precision and depth in nutrient estimation. This contrasts with the LSTM model's tendency to favor frequently occurring ingredients, underlining the benefits of multimedia training in improving nutrient estimation accuracy.

### 2.3 UMDFood‐VL Model Accelerates Chemical Analysis and Validated by Chemical Experiment

**Figure 4:**
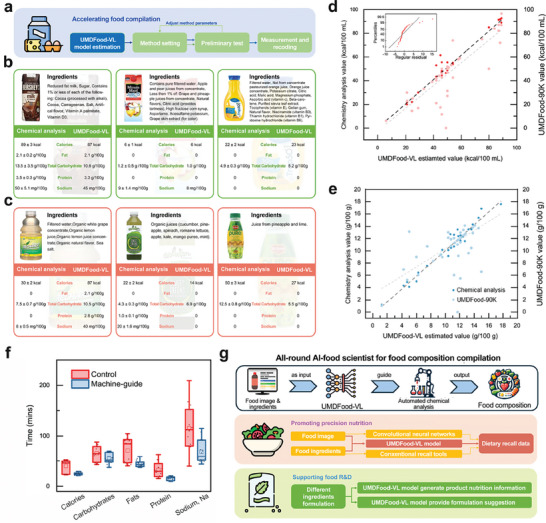
Validation and Application of the UMDFood‐VL Model through Chemical Analysis. a) A schematic representation of the UMDFood‐VL model's role in expediting the compilation of food composition data. b, c) Alignment between UMDFood‐VL model‐estimated values and chemical analysis ground truth values for selected beverage samples, with examples of either accurately estimated (B) or inaccurately estimated samples c). d) Correlation between calorie values estimated by the UMDFood‐VL model and those derived from chemical analysis, in comparison to the UMDFood‐90k dataset, with an inset showing residual analysis plot (n = 50). e) Correlation analysis for carbohydrate estimations, juxtaposing UMDFood‐VL model‐estimated values against chemical analysis ground truth and UMDFood‐90k database values (n = 50). f) A comparison of the time required for macro‐nutrient analysis using conventional methods.^[^ ^4^ ^]^ versus the machine‐guided approach facilitated by the UMDFood‐VL model, with participant teams split into two groups for the analysis (n = 30). g) Outlines the integration of the UMDFood‐VL model into a comprehensive AI food scientist system, which utilizes the model for recognizing food samples and providing data for automated chemical analysis procedures. H) Flow charts detailing the potential applications of the UMDFood‐VL model in enhancing precision nutrition and food research and development (R&D).

To evaluate the efficacy of the UMDFood‐VL model in accelerating the analysis of chemical composition, we executed an empirical study using a set of 50 random beverage samples (**Figure** **4**a). This category was specifically chosen to mirror the contemporary trend toward innovative and diverse food products, highlighting the complexities and variabilities inherent in composition compilation for such items. It was posited that food scientists could leverage the UMDFood‐VL model's estimated values as provisional equivalents to preliminary experimental values.

Our analysis revealed that for five critical nutrients—calories, carbohydrates, proteins, fats, and sodium—82.2% of the beverage samples exhibited a UMDFood‐VL model estimation error within a 10% margin of their chemical analysis ground truth values. To delve deeper, we examined six representative samples, divided equally between those accurately estimated by the model and those less so, to compare model‐estimated values against chemical analysis ground truth (Figure 4b,c). For the accurately estimated samples, there was a significant congruence between the model's predictions, chemical analysis data, and existing database records, demonstrating the model's capacity to meet required accuracy benchmarks. Conversely, the samples exhibiting substantial estimation discrepancies underscored the model's limitations, particularly with unique or unconventional products, such as a green yogurt beverage—an outlier within the beverage category—and pineapple and lime juice, which deviated from the typical caloric range for similar products.

Highlighting the comparative effectiveness of the UMDFood‐VL model, we observed a higher correlation between the chemically analyzed values and the model‐estimated values for calorie content (with an *r^2^* of 0.97 for UMDFood‐VL versus 0.68 for UMDFood‐90K). Residual analysis, considering chemically analyzed values as the ground truth, showed a marked decrease in outliers among the predictions made by the UMDFood‐VL model compared to values derived from the UMDFood‐90K dataset (Figure 4d). This was particularly pronounced in sodium content analysis, where the correlation coefficients (*r^2^*) were 0.96 for UMDFood‐VL and 0.24 for UMDFood‐90K, suggesting an error‐correction capability within the UMDFood‐VL model for database inaccuracies (Figure 4e).

To further ascertain the UMDFood‐VL model's contribution to enhancing the efficiency of food chemical analysis, we engaged 30 teams from a food composition analysis laboratory class in a controlled experiment. The teams were evenly divided, with one group receiving UMDFood‐VL model estimates as a reference before conducting their experiments. The findings indicated that the group with access to the model's estimates experienced a significant reduction in time spent on the experiments, with an average decrease of 36.9%, thereby underscoring the model's potential to streamline and expedite the food analysis process (Figure 4f).

## 3 Discussion

The assembly of composition information for foods products is an intricate and labor‐intensive endeavor.^[^ ^7^, ^22^ ^]^ To effectively harness the abundant, albeit noisy, data available, the deployment of multi‐modal modeling, particularly VLM, is imperative.^[^ ^23^ ^]^ Our methodology utilizes vision‐language models to concurrently extract profound insights from both food imagery and ingredient statement texts. These models serve as a potent analytical tool, capable of parsing and extracting features from sparse and noisy datasets without necessitating arduous preprocessing of text and images. Their robustness against data imperfections is noteworthy. Crucially, language models facilitate the quantization of nutrition representation without the prerequisite of quantitative data on each ingredient, which is often not readily available due to commercial constraints. This is accomplished through the precise alignment of information intersected between images and texts, and by collaboratively leveraging complementary information to achieve a holistic understanding of food samples. The UMDFood‐VL estimator thus enables machines to perceive, interpret, comprehend, and analyze multi‐media food data, ultimately yielding accurate nutrient estimations.

Traditional vision models, such as CNN, encounter difficulties with packaging images of food products due to the presence of extraneous information that impedes nutrient estimation.^[^ ^21^, ^24^ ^]^ For instance, the depiction of strawberries may be common to both strawberry‐flavored milk and juice, potentially leading CNN models to erroneously prioritize the strawberry icon and bias the prediction outcomes. By integrating ingredient statements, more definitive features can be discerned from the package image and vice versa, culminating in enhanced accuracy (see food nutrient estimation model summary in Table S2, Supporting Information). The UMDFood‐VL estimation methodology satisfies the precision demands of current food composition compilation tasks, employing a lenient acceptance range for discrepancies between database and chemical values to adhere to regulatory standards. For nutrients like fat, a tolerance threshold of less than 120% is maintained, while beneficial nutrients such as vitamins, protein, and dietary fiber must exceed baseline nutritional label values by at least 80%. When calorie data estimates fall below 120% of the dataset's verifiable ground truth, a substantial 90.74% of products are classified as accurately estimated (Tables S3–S6, Supporting Information).

Moreover, the UMDFood‐VL model pioneers a novel methodology for food analysis. It is the pivot of a system of AI food scientists, with the potential for autonomous analysis like other AI robot chemists.^[^ ^25^ ^]^ Besides, by scrutinizing the weights allocated to various ingredients, a correlation between specific ingredients and nutrients is established. For example, in estimating sodium content, not only were food additives containing sodium accorded high significance, but ingredients such as “cheese,” “wheat,” and “yeast” were also emphasized, suggesting a predisposition for higher sodium content in bakery products. The model also facilitates personal dietary record‐keeping, enabling users to rectify uncertain items recognized by machine image analysis through ingredient selection. Additionally, it aids in the development of new products by allowing for the quantitative analysis of changes in model‐extracted feature vectors when altering color ingredients, thereby identifying viable substitutes (Figure 4h).

Despite these promising outcomes, the UMDFood model is not without its limitations. Primarily, while our self‐supervised pre‐training techniques based on the CLIP model enhance labeling efficiency, the approach still necessitates expert‐annotated training data for supervised fine‐tuning. Consequently, the model is limited to predicting nutrient values explicitly annotated in the dataset and is unable to forecast unannotated nutrients, such as phytochemicals. Furthermore, the model's efficacy is contingent on the availability of extensive multi‐media food data, the compilation of which is a formidable task necessitating considerable time and effort. The training of our model also demands substantial computational resources and time investment. Moreover, while the current focus is on food imagery and ingredient texts, incorporating additional modalities like taste and chemical quantization could further augment the model's accuracy through joint learning. Expanding the database to include food sensory and safety information would broaden the model's applicability, facilitating the advancement of precision nutrition and heralding a new epoch in the food industry. Future research endeavors will explore self‐supervised learning methodologies that obviate the need for extensive labeled food data, aiming to expand the content of the food multimodal database and enhance model performance. In addition, while the analytical methods used in this study are standard, we recognize that there are more advanced techniques available, such as Inductively Coupled Plasma Optical Emission Spectrometry (ICP‐OES), which offer higher accuracy. Our study focuses on demonstrating the integration of VLM to enhance the efficiency of nutritional analysis. Future work will aim to incorporate state‐of‐the‐art analytical methods to further improve the accuracy and reliability of nutritional value estimation.

## 4 Conclusion

In conclusion, UMDFood‐VL emerges as a groundbreaking VLM, adept at swiftly estimating essential nutrients from food product images and labels, catering to the urgent demands for precise nutrition information across clinical, precision nutrition, and food industry sectors. Harnessing the power of the extensive UMDFood‐90k database, the model exemplifies unparalleled accuracy, achieving a macro‐AUCROC of up to 0.921 in fat estimation and accurately aligning with chemically analyzed ground truth for 82.2% of products within a less than 10% deviation margin. Notably, UMDFood‐VL markedly reduces the time required for experimental analysis by 36.9% when tested amongst students, significantly boosting the efficiency of nutritional data gathering. This model not only facilitates a more streamlined approach to nutrient estimation but also establishes new benchmarks for accuracy and utility in food‐related domains. The introduction of UMDFood‐VL represents a seminal advancement in the field, promising to transform the landscape of nutritional data analysis and its application, thereby meeting and exceeding the current and future needs of the food industry and nutritional science.

## 5 Experimental Section

### Datasets

*Training*: The method was trained on the proposed UMDFood‐90k dataset, containing 89533 food product samples. Each sample consists of an ingredient statement, and product image, and the values of 5 nutrients including fat, protein, sodium, calories, and carbohydrates. The nutrition value were considered estimation task as a multi‐classification task, converting the continuous values of each nutrient into discrete classification labels. For each nutrient, the 0.95 percentile was first chose as the threshold and filtered out food products that have higher nutrient values than the threshold to avoid the effect of outliers. Then, the zero value were marked as class 0, sorted other non‐zero nutrient values in ascending order, and evenly separated them into different classes. To keep the data balanced, the number of classes for these non‐zero nutrient values depended on the multiple non‐zero nutrient values to zero nutrient values. In the end, there were 4 classes for fat, 6 classes for protein, 8 classes for protein, 38 classes for calories, and 18 classes for carbohydrates. Details of the UMDFood‐90k dataset were in the supplementary material.

*Evaluation*: The whole dataset was split into a training set and a testing set in a 7:3 ratio. The testing dataset was leveraged to calculate the macro‐averaging Area Under the Receiver Operating Characteristic Curve (macro‐AUCROC) which was commonly used in the evaluation of binary classification tasks. To adapt these metrics to the multi‐classification task, the One‐versus‐One (OvO) strategy was leverage that considers all possible pairwise combinations of classes. For each round, the strategy considers one class as the positive class and another class as the negative class and discards all the others. In this way, the multi‐classification task was reduced output into a binary classification one and calculate the AUC score. By leveraging the macro‐averaging, the unweighted mean of AUCROC scores of all pairwise combinations were taken as the macro‐AUCROC. By leveraging the weighted averaging, the average of AUCROC scores of all pairwise combinations weighted by the number of true positive instances were taken for each pairwise combination.

*Pre‐processing*; Each image was resized to 224 × 224 with 3 (RGB) channels. For each ingredient statement, removed special characters and punctuations were lowercased and tokenized it using a byte pairing encoding scheme with a vocabulary size of 49408. The maximum token sequence length was set to 77, and the text embedding was truncated if the text embedding of the ingredient statement exceeds the maximum length.

### Architecture for Two Solutions

The UMDFood‐VL model was constructed on top of the CLIP model for the food nutrient estimation tasks (Figure 2b). In specific, the UMDFood‐VL model contains a CLIP model and an MLP classifier. The CLIP model has an image encoder to handle image data and a text encoder to process text descriptions. The Vision Transformer which has a 32×32 input patch size was used, 12 attention layers, and 12 attention heads. The text encoder was a Transformer with 12 attention layers and 8 attention heads. The MLP classifier takes image embeddings, text embeddings, or both of them as input and the ground truth nutrient value as output. The MLP classifier contains 3 layers, and the number of neurons in each layer was set to 64, 16, and the number of classes for the given nutrient.

### Implementation

*Pre‐Training*. The pre‐trained CLIP officially released by Open AI was used.^[^ ^13^ ^]^ The pre‐training procedure of CLIP was conducted on YFCC100M dataset, a publicly available dataset that contains image‐caption pairs, using self‐supervised learning, i.e., natural language supervision.^[39]^

*Training*: Two solutions were provided to construct the UMDFood model. In the first solution, the CLIP model was fixed as a feature extractor and only the MLP classifier was optimized by cross‐entropy loss on the UMDFood‐90K dataset. The UMDFood model was refer to in this solution as UMDFood‐E. Adam as the optimizer was leveraged with a learning rate of 1e‐3. All MLP classifiers had a batch size of 128 and were trained for 100 epochs. This solution was further categorized by the input data. Specifically, “V” stands for input from the visual modality, i.e., product images, and “L” stands for input from the language modality, i.e., ingredient statements. To prepare the input data for training the MLP classifiers, the ingredient statements were first fed and product images into the CLIP model to get the text embeddings and image embeddings, respectively. The MLP classifiers of UMDFood‐VF, UMDFood‐LF, and UMDFood‐E‐VL take the image embeddings, text embeddings, and the concatenation of both image and text embeddings as input, respectively. In the second solution, the CLIP model was finetuned while training the MLP classifier, so both the CLIP model and MLP classifier were optimized by cross‐entropy loss on the UMDFood‐90k dataset. The UMDFood model was refer to in this solution as UMDFood‐VL. Adam as the optimizer was leveraged with a learning rate of 1e‐3 for the MLP classifier and 1e‐7 for the CLIP model. All models in the second solution had a batch size of 128 and were trained for 100 epochs. The UMDFood‐V, UMDFood‐L, and UMDFood‐VL take the product images, ingredient statements, or both as input.

### Chemical Analysis

*Materials* Sodium carbonate (99.5%), benzoic acid (99.5%), petroleum ether, potassium chromate (99.5%), silver nitrate (99.0%), phenol (98%), concentrated sulfuric acid (96%) were all purchase from Sigma‐Aldrich (St. Louis, MO, USA). All chemicals were of analytical grade.

*Sample Preprocessing*: Weighed 500 g of all beverage samples and then freeze‐dried them before subsequent analysis. Record weight after freeze‐dried as W_f_.

*Calorie Determination by Bomb Calorimetry*: Weighted out 0.5 g of sample, place it into the holder, and fit it into the pellet press. Then pull down the lever of the pellet press three times and record the value. Calories was measured by the Parr 6400 bomb calorimeter which was loaded with oxygen automatically and the change in the temperature was measured and logged by the control computer. Benzoic acid was used as the calibration standard with GE of 6.318 kcal/g.

*Total Carbohydrate Determination by Phenol Sulfuric Acid*: Weighed 100 mg of samples into a test tube and heat block at 100 °C for 3 h with 5 mL of 2.5 N HCl and cool to room temperature. Then neutralized with 660 mg solid NaCO_3_ until the effervescence ceases. Transfer 2.5 mL of sample solution to a centrifuge tube and make up the volume to 50 mL, vortex mixing for 1 min. Subsequently, filtrate the sample solution with filter paper, and collect 2 mL of the final sample solution into a centrifuge tube. Add 5 mL of 96% sulfuric acid to each tube rapidly and cool down the tubes under a fume hood for 10 min. Read the absorbance at 490 nm for each sample and calculate by standard curve (R^2^ = 0.9992)

*Protein Determination by Combustion*: First, 1.0 ± 0.2 g of post‐freeze‐dried samples were weighed in a tin boat. Analyze at least five blanks with tin boats, until three consecutive blanks have a stable value with a standard deviation of less than 0.002%. Blank correct using the last three consecutive values. Load the set of samples into the protein analyzer. Instrumental settings including the furnace temperature of 1100 °C, and purge cycles were twice. The baseline delay time for 6 s, the minimum analysis time for 35 s equilibrate time for 30 s, and T_C_ baseline time for 10 s. The nitrogen factor was set to 6.25.

*Fat determination* First, accurately weigh an extraction flask and then add approximately 85 mL of petroleum ether. Then weighted 4.0 g of samples to a thimble. Soxhlet extraction for at least 50 cycles in a minimum of 4 h. Upon completion of extraction, separate the unit and pour off the ether from the Soxhlet extraction apparatus into a large filter positioned on a bottle. Disassemble the Soxhlet apparatus, then put the flask in a steam bath to vaporize the residual petroleum ether until the weight is constant. The fat content of sample C_Fat_ was calculated by Equation 1

(1) $$C_{\mathrm{Fat}}=0.05\left(W_{a}-W_{b}\right)W_{f}$$

where C_Fat_ was fat content (g/100 g), W_a_ was the weight of the flask after extraction (g), W_b_ was the weight of flask prior to extraction (g).

*Sodium Determination by Mohr titration*: Pipette accurately 10.00 g of beverage drink samples before freeze‐drying in duplicate into 250 mL Erlenmeyer flasks. Added 40 mL of boiling water to each Erlenmeyer flask and stir the mixture vigorously for 1 min. Then 1 mL of K₂CrO₄ indicator was to each 50 mL of the prepared sample. Titrate each solution with standardized 0.1 M AgNO_3_ (0.1000 ± 0.0005 M) to the first visible pale red‐brown color that persists for 30 seconds. The sodium content of the sample was calculated by Equation 2

(2) $$C_{\mathrm{Sodium},\;\mathrm{Na}}=39.07\;V_{\mathrm{Ag}+}$$

where C_Sodium, Na_ was the content of sodium (g/100 g), V_Ag+_ was the volume of standardized AgNO_3_ volume titrant used (mL).

## Conflict of Interest

The authors declare no conflict of interest.

## Author Contributions

P.M., Y.W. and N.Y. carried out the concept and designed the experiments. P.M. collected and reviewed the UMDFood‐90k dataset. P.M. and Y.W provide coding and analyze the result. X.J. and Y.H participated in the experiment. P.M., Y.W., and N.Y. collectively wrote the paper. Y.Z., M.B., Q.W., and C.W. supervised the study, participated in experiments design, coordination and helped to draft the manuscript. All authors commented on the final manuscript.

## Supporting information

Supporting Information

## Data Availability

The data that support the findings of this study are available from the corresponding author upon reasonable request.
