# Supplementary material for: Integrating Vision‐Language Models for Accelerated High‐Throughput Nutrition Screening
Source: Adv Sci (Weinh). 2024 Jul 8;11(34):2403578. doi: 10.1002/advs.202403578 (PMC11425866; doi:10.1002/advs.202403578)
Supplement: Supplementary file 1 — Supporting Information [file ADVS-11-2403578-s001.pdf]

ADVANCED SCIENCE
Open Access

# Supporting Information

for *Adv. Sci.*, DOI 10.1002/advs.202403578

Integrating Vision-Language Models for Accelerated High-Throughput Nutrition Screening

*Peihua Ma, Yixin Wu, Ning Yu, Xiaoxue Jia, Yiyang He, Yang Zhang, Michael Backes, Qin Wang and Cheng-I Wei**

# Supporting information

## Integrating Vision-Language Models for Accelerated High-Throughput Nutrition Screening

**Peihua Ma[1], Yixin Wu[2], Ning Yu[3], Yang Zhang[2], Xiaoxue Jia[1], Yiyang He[1], Michael Backes[2], Qin Wang[1], Cheng-I Wei[1,*]**

1. Department of Nutrition and Food Science, College of Agriculture and Natural Resources, University of Maryland, College Park, MD 20742, United States
2. CISPA Helmholtz Center for Information Security, Saarbrucken, Germany
3. Netflix Eyeline Studios, Los Angeles, CA 90028, United States

***Email:** wei@umd.edu

# Content

**Section 1: UMDFood-90k database**

Figure S1 Supporting characteristic of UMDFood-90k dataset

Figure S2 Heat map of ingredients frequency in different categories

Table S1 UMDFood-90k data range and units

**Section 2: Model structure**

Figure S3 Flow chart of UMDFood-VL model

**Section 3: Training metrics**

Figure S4 Macro-AUCROC curve of different model for different nutrients

Figure S5 Estimation value error distribution of different models for different nutrients

Figure S6 Training accuracy, testing accuracy, training loss, and testing loss in the training process

Figure S7 Macro-F1, micro-F1 and Macro-AUCROC in the training process

Table S2 Training log from server

Table S3 Training metrics of different models

Table S4 Supporting training metrics of different UMDFood model (part 1)

Table S5 Supporting training metrics of different UMDFood model (part 2)

**Section 4 Comparison between different food categories among UMDFood-90k**

Figure S8 Calories estimation results of different food categories in UMDFood-90k

Figure S9 Fat estimation results of different food categories in UMDFood-90k

Figure S10 Carbohydrate estimation results of different food categories in UMDFood-90k

Figure S11 Protein estimation results of different food categories in UMDFood-90k

Figure S12 Sodium estimation results of different food categories in UMDFood-90k

Figure S13 Confusion matrix of different models for different nutrients

**Section 5 Chemical analysis result of beverage products**

Figure S14 Comparison of nutrient value between USDA-BFPD, UMDFood-VL and chemical analysis value.

**References**

## Section 1. UMDFood-90k database

Here, we provide further details about the UMDFood-90k database that was collected. Initially, we examined the distribution of ingredient list lengths and represented the cumulative frequencies of various lengths (**fig. S1a)**. The findings revealed that, on average, each product in the UMDFood-90k database contains approximately 13 ingredients. Moreover, for food items with 20 or fewer ingredients, the distribution of ingredient quantities appeared to be uniformly distributed, as indicated by the nearly linear cumulative frequency.

In **fig. S1b**, we present the top 20 categories within the UMDFood-90k database. These categories were classified following the criteria inherited from the BFPD database. The most prominent category was "candy," with 12,462 items collected. Following that were "cheese" and "popcorn, peanuts, seeds, and related snacks" with 12,018 and 11,298 items, respectively. It's important to note that in this manuscript, we merged several categories into larger ones for the purpose of discussion. Among the databases, the largest category was beverages, including a total of 19,428 items, which encompassed "fruit and vegetable juice, nectar and fruit drinks," "yogurt," "soda," and "water."

Additionally, we analyzed the frequency of occurrence of components in the database, as depicted in **fig. S1c**. We tokenized the ingredient statements by commas or other special punctuation marks and manually reviewed them. The three most frequently appearing components in the UMDFood-90k database were "salt," "sugar," and "water," with 94,557, 82,235, and 73,560 occurrences, respectively. We recorded the frequency of more than 10,000 occurrences for 50 components. It was observed that there was a clear long-tail distribution, where the number of occurrences for many ingredients was insignificant compared to the top

components. Consequently, encoding the data using one-hot vector or TF-IDF methods often resulted in a sparse database, limiting the effectiveness of machine learning. It's important to acknowledge that ingredient statistics are repetitive and may not fully represent the ingredient list due to the lack of accurate tools for tokenizing food ingredient lists. For instance, the existence of "corn starch," which may be divided into "corn" and "starch" during the word tokenization process, cannot be entirely excluded. We are currently developing a more precise food ingredient statement tokenization tool based on the GPT-4 model. However, these statistics do not impact the main content of our study in this article, which primarily focuses on the use of the UMDFood model and the evident long-tail distribution of food ingredients in UMDFood-90k.

Furthermore, we analyzed the occurrences of ingredients across different food categories and observed a clear correlation, as shown in **fig. S2**. For instance, "salt" appeared most frequently in the "cheese" category, partly due to the high number of cheese products in the UMDFood database and partly because a higher proportion of cheese products contain salt. Ingredients can be divided into two types based on their relationship with food categories. The first type includes ingredients highly related to specific food categories, such as "juice," which predominantly appears in the "fruit and vegetable juice, nectar and fruit drinks" category. The second type comprises ingredients that are not strongly associated with any specific food category, such as "salt," "sugar," and "oil." Due to the complexity of this correlation, conventional models often struggle to provide satisfactory results during fitting.

During the process of collecting the database, we eliminated extreme values from the BFPD based on the nutrient distribution range. The specific criteria for including products are outlined in **Table 1**. It is worth noting that less than 1% of the total data volume was excluded based on

these criteria. To facilitate more efficient training, we grouped all nutrients based on a certain interval. For instance, group number 0 represented products with 0 kcal, while group number 1 represented products with 30 kcal/100 g. The model training demonstrated significant improvement compared to the uncoded regression training group, as indicated in Table S4 (refer to section 3 for more details).

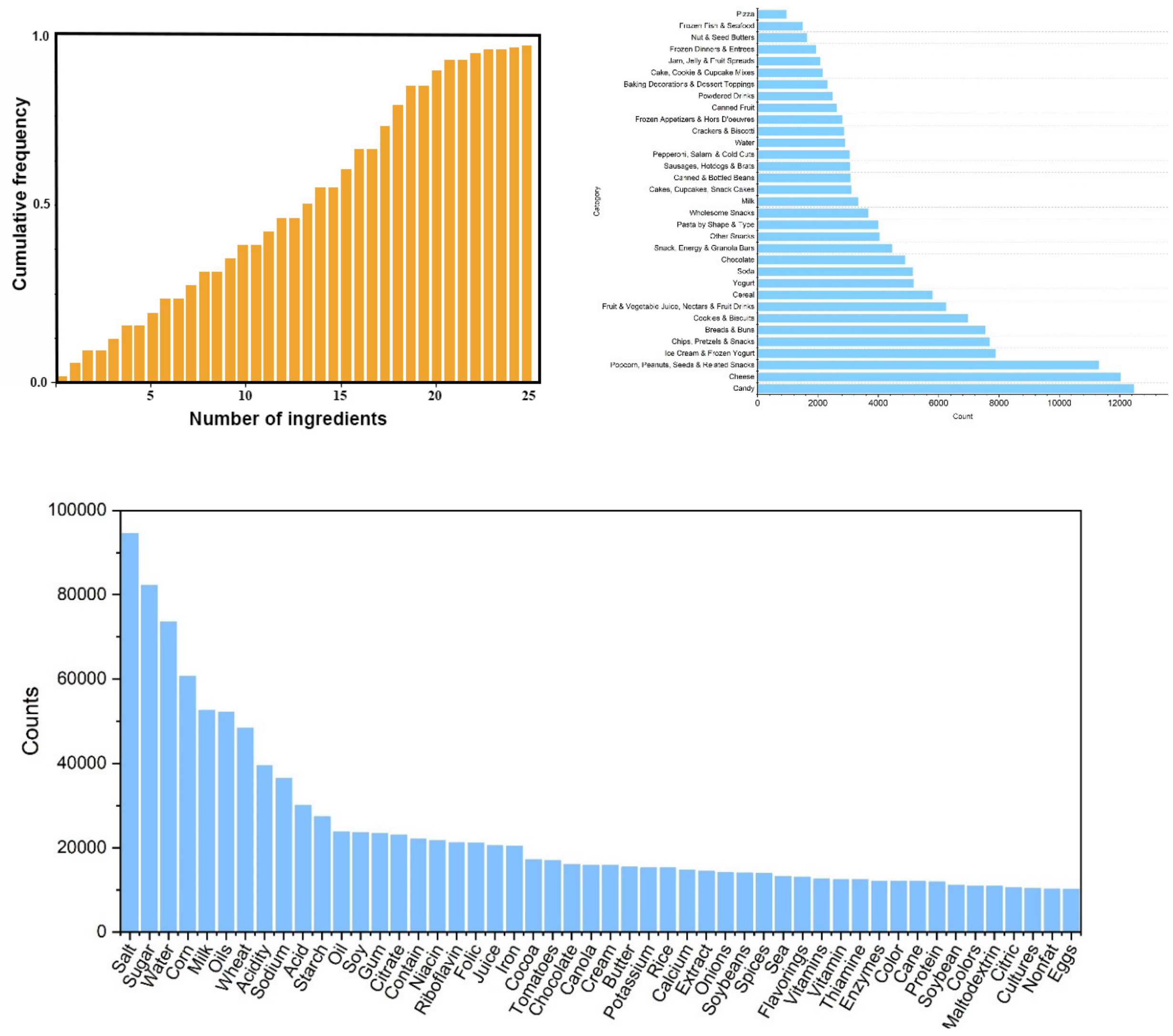


**Figure S1 Supporting characteristic of UMDFood-90k dataset** a, the distribution of the number of ingredients in each item among UMDFood-90k; b, the category distribution of UMDFood-90k; c, the distribution of the ingredients among UMDFood-90k.

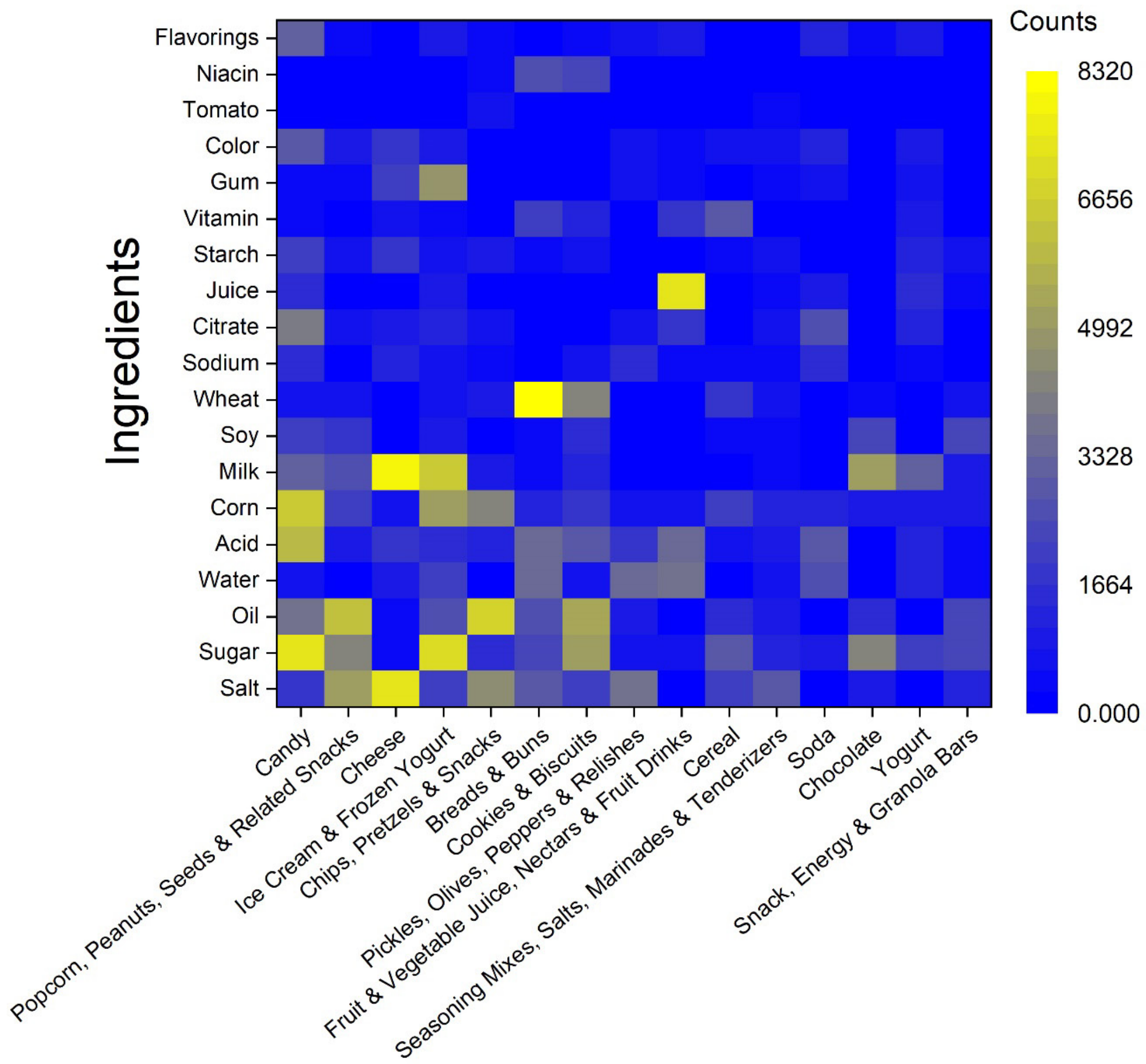


**Figure S2** Heat map of ingredients frequency in different categories.

**Table S1 UMDFood-90k data range and units**

| | Range | Units | Category bin width |
|---|---|---|---|
| Calories | 0-1000 | kcal/100g | 30 kcal/100g |
| Protein | 0-100 | g/100g | 2 g/100g |
| Total carbohydrate | 0-100 | g/100g | 5 g/100g |
| Fat | 0-100 | g/100g | 2 g/100g |
| Sodium, Na | 0-3000 | mg/100g | 60 mg/100g |

**Table S2 Summary of food nutrient estimation model**

| Dataset | Total# images | Total# class | Data type | Accuracy |
|---|---|---|---|---|
| FoodLog | 6512 | 2000 | Images with calorie values | 79% of the estimations are correct within ±40% error and 35% correct within ±20% error. |
| Menu-match | 646 | 41 | Images with tags and calorie values | average recall rates of 83% or 92% for a list of 5 food items |
| FoodDD | 3000 | 30 | Images with categories | 100% accuracy |
| Inselspital dataset | 1620 | 248 | Images with categories, bounding boxes, actual volume and CHO grams | 89.48% accuracy |
| Okamoto | 120 | 20 | Images with calorie values | 52.23% average absolute errors |
| Chokr | 1132 | 5 | Images with categories, size and calorie values | N/A |
| MADiMa | 21807 | 80 | Images with depth maps, weight, volume and nutrient composition | 97.7% accuracy |
| Fast food | 20 | 14 | Image pairs with categories and nutrient composition | 98.8 accuracy |
| VIPER | 14991 | 82 | Images with categories and bounding boxes | N/A |
| ChinaFood-100 | 10047 | 100 | Images with nutrient composition | 96.62% in top-5 accuracy |
| NIAD | 1281 | 521 | Images with depth, recipes and nutrient composition | 91% accuracy |
| ChinaMartFood-109 | 10921 | 18 | Images with nutrient composition | 78 % and 94 % for top-1 and top-5 accuracy |

## Section 2 Model structure

Our classification model comprises an image encoder, a text encoder, and a MLP classifier (**fig. S3**). The image encoder takes a product image as input, while the text encoder processes the ingredient statement. The MLP classifier combines the image and text embeddings to make the final decision.

In the image encoder, the input image is initially split into a grid of non-overlapping 32x32 patches, each with 768 channels. Each patch can be viewed as a token with 768 dimensions. These patches are then flattened into vectors and projected into lower-dimensional patch embeddings. Additionally, a class embedding, and a position embedding are added to each patch embedding. These learned embeddings allow the model to incorporate information about the image's classification label and capture the spatial relationships between the patches. Layer normalization is applied to normalize feature activations within a single training example.

The patch embeddings are then passed through a series of residual Transformer blocks. Each block consists of a 12-head self-attention mechanism and a feedforward neural network. The self-attention mechanism enables the model to focus on different parts of the input sequence, while the feedforward neural network learns non-linear relationships between the patch embeddings. The final projection layer maps the output of these Transformer blocks to the target output space, enabling predictions to be made for the input images.

The text encoder is also a transformer-based neural network. It takes a sequence of text tokens as input, which are initially embedded into fixed-length token embeddings. Position embeddings are added to the token embeddings to capture sequential dependencies between the tokens and enhance the representation of the input text. The token embeddings are then processed through

12 transformer blocks. Each block incorporates a multi-head self-attention layer to capture contextual relationships between the tokens and a feedforward neural network to transform the feature representation. Layer normalization is applied after each block to stabilize the training process. The final projection layer produces a vector representation of the input text that captures its overall semantic meaning.

Once the image embeddings from the image encoder and the text embeddings from the text encoder are obtained, the 3-layer MLP classifier learns a function that maps the combined embeddings to output predictions. This classifier combines information from both modalities to make accurate predictions.

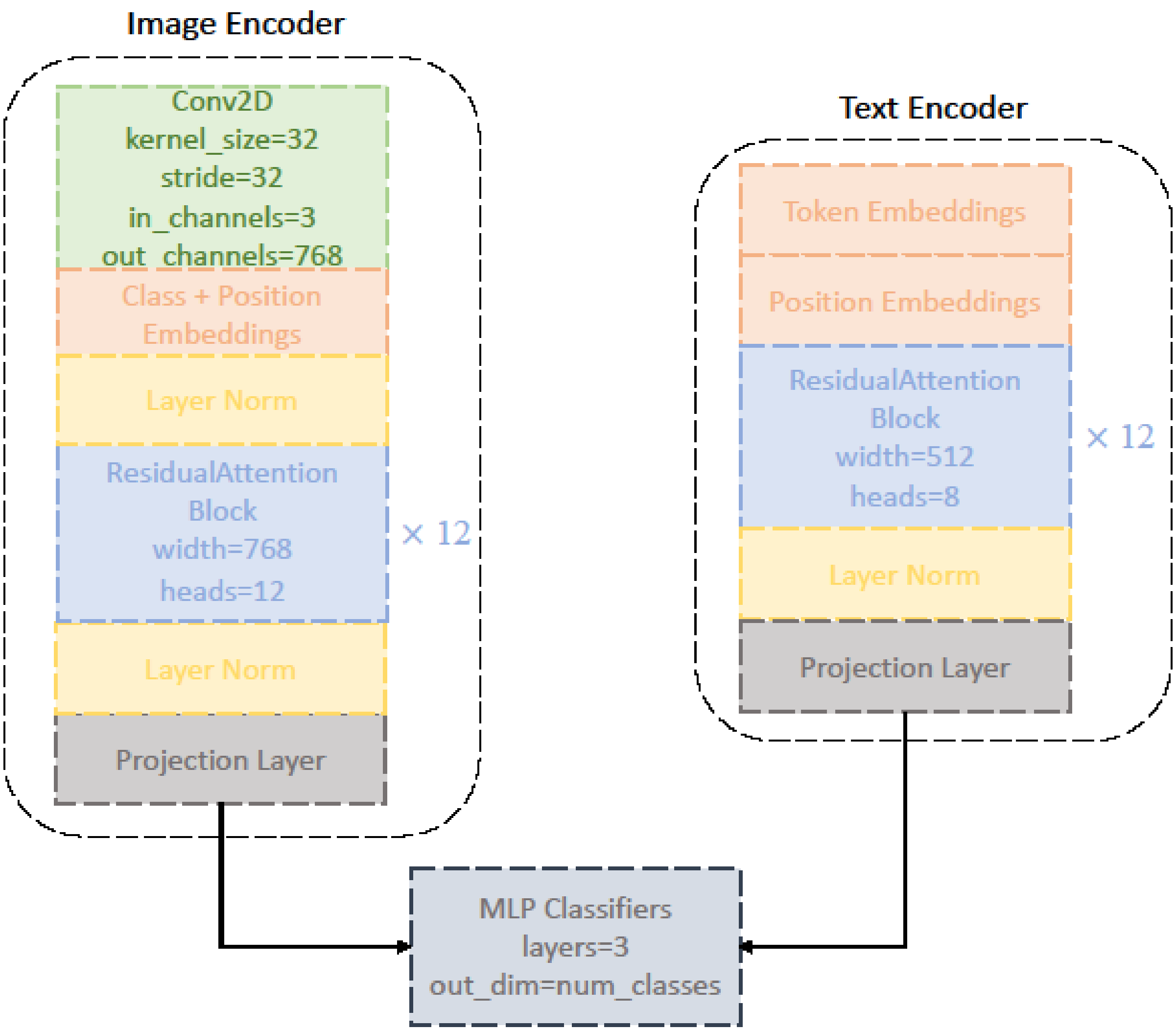


**Figure S3** Flow chart of UMDFood-VL model

## Section 3 Training metrics

The ROC (Receiver Operating Characteristic) curve is a widely used metric for evaluating the performance of classification models. It provides a graphical representation of the trade-off between the false positive rate (FPR) and the true positive rate (TPR) at different threshold settings. The FPR, plotted on the x-axis, represents the proportion of negative samples that are incorrectly classified as positive. The TPR, plotted on the y-axis, represents the proportion of positive samples that are correctly classified as positive. The AUC (Area Under the Curve) score measures the overall performance of the model and ranges from 0 to 1. A perfect classifier achieves an AUC score of 1, while an AUC score of approximately 0.5 indicates a model with no discrimination capacity or random guessing. An AUC score approaching 0 suggests that the model is inversely classifying the classes. In the case of multi-class classification, the ROC curve is obtained by reducing the problem to binary classification using the One vs One (OvO) strategy, which compares all possible combinations of two classes. The AUC score is reported in the main text, and the ROC curve is provided in the appendix (**fig. S4**) to visualize the trade-off between FPR and TPR and aid in selecting an optimal decision threshold.

In our study, it is evident from the ROC curve that the areas under the curve of UMDFood-VL for different nutrients are much larger compared to UMDFood-L and UMDFood-V, indicating superior performance.

In the main text, we present the model training results in terms of error distributions, which are grouped into intervals of 20% for easier interpretation in the food sector (**fig. S5**). We observe that different nutrients have varying effects on the model's prediction results. Due to variations in data distribution for each nutrient, the model's performance also varies. For instance, the model

shows better fitting for calorie content, as it follows a normal distribution. Conversely, the model exhibits the most significant improvement in predicting fat and protein values. Compared to a purely visual model, the number of predictions for fat values below 20% doubles, highlighting the insufficient information in the visual-only approach for effective training and learning. This observation aligns with the findings from the ROC curve. However, the model's improvement in predicting sodium content is not as substantial, likely due to the significant deviation of sodium data distribution from a normal distribution. This suggests limitations in the current model for this specific type of data.

Furthermore, as described in the main text, UMDFood-VL demonstrates significant improvement over other models in terms of test macro-AUCROC and weighted-AUCROC. **Fig. S6** provides additional insights by displaying the training accuracy, testing accuracy, training loss, and testing loss during the training process. The x-axis represents the optimization step. While other models may exhibit higher training accuracy and lower training loss during training, the evaluation results consistently show that UMDFood-VL achieves the highest test accuracy and lowest test loss. This indicates that UMDFood-VL has better generalizability for nutrient estimation tasks. Supplementary results including Micro-F1, Macro-F1, and Macro-AUCROC on the test set also support these findings (**fig. S7**).

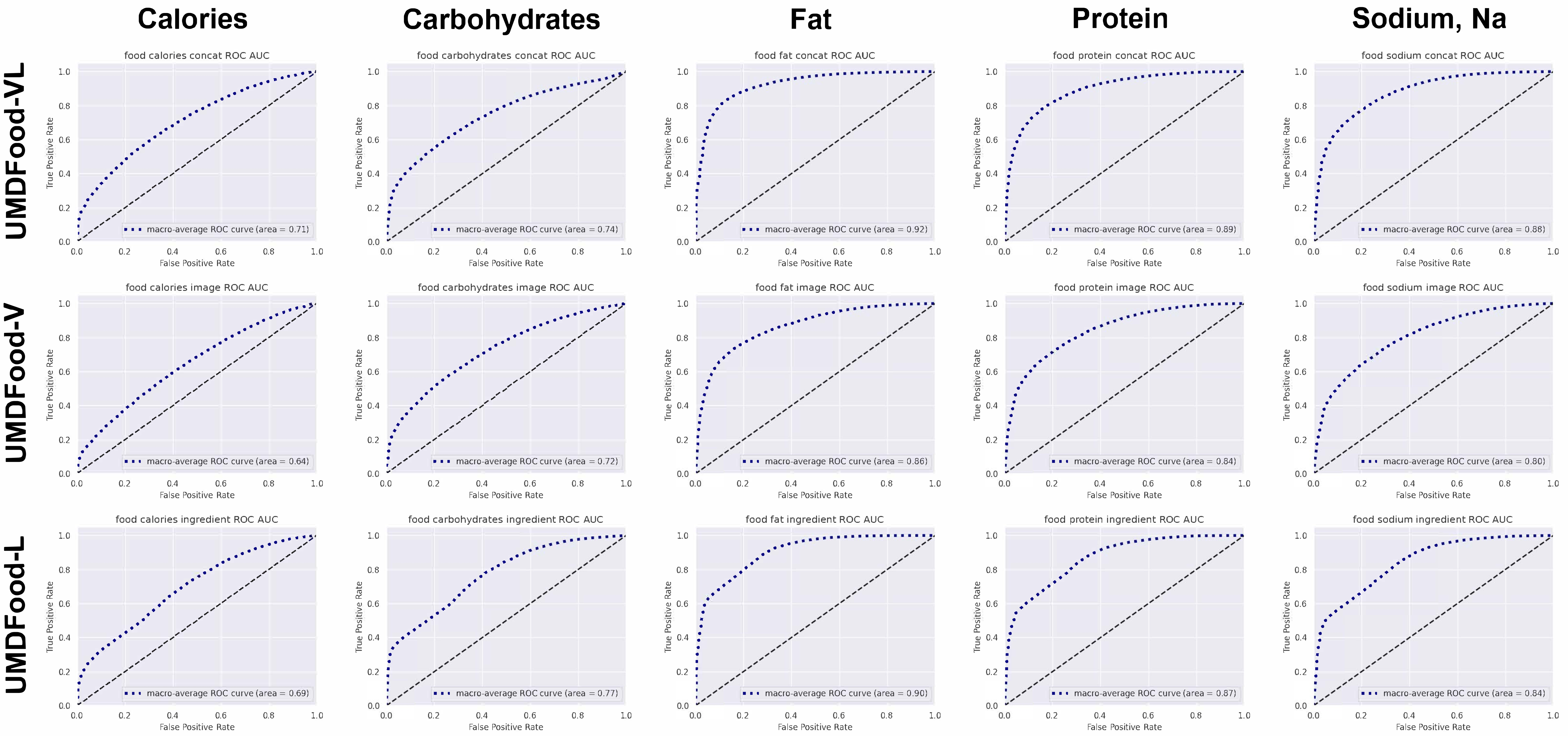


**Figure S4** Macro-AUCROC curve of different model for different nutrients

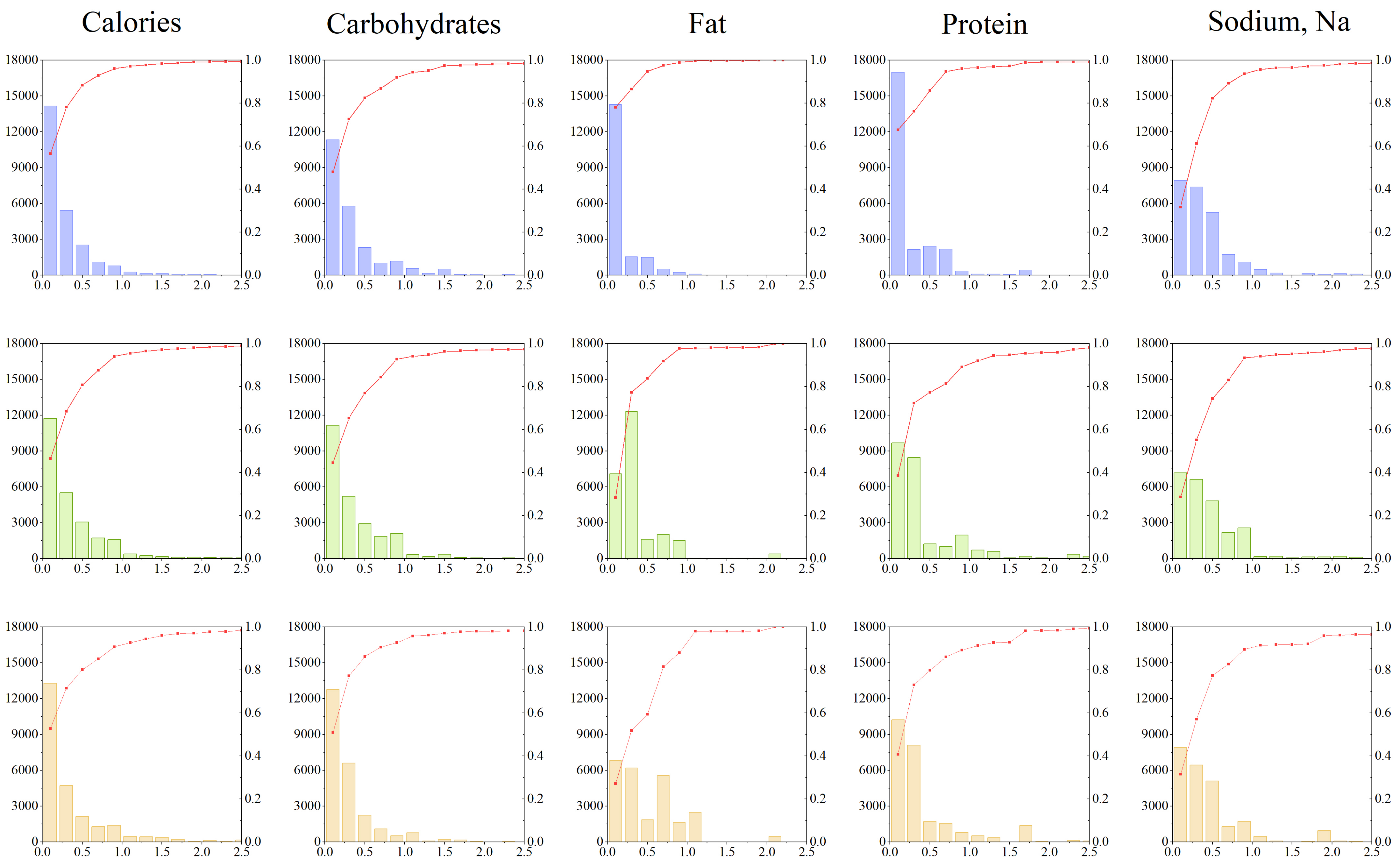


**Figure S5** Estimation value error distribution of different models for different nutrients

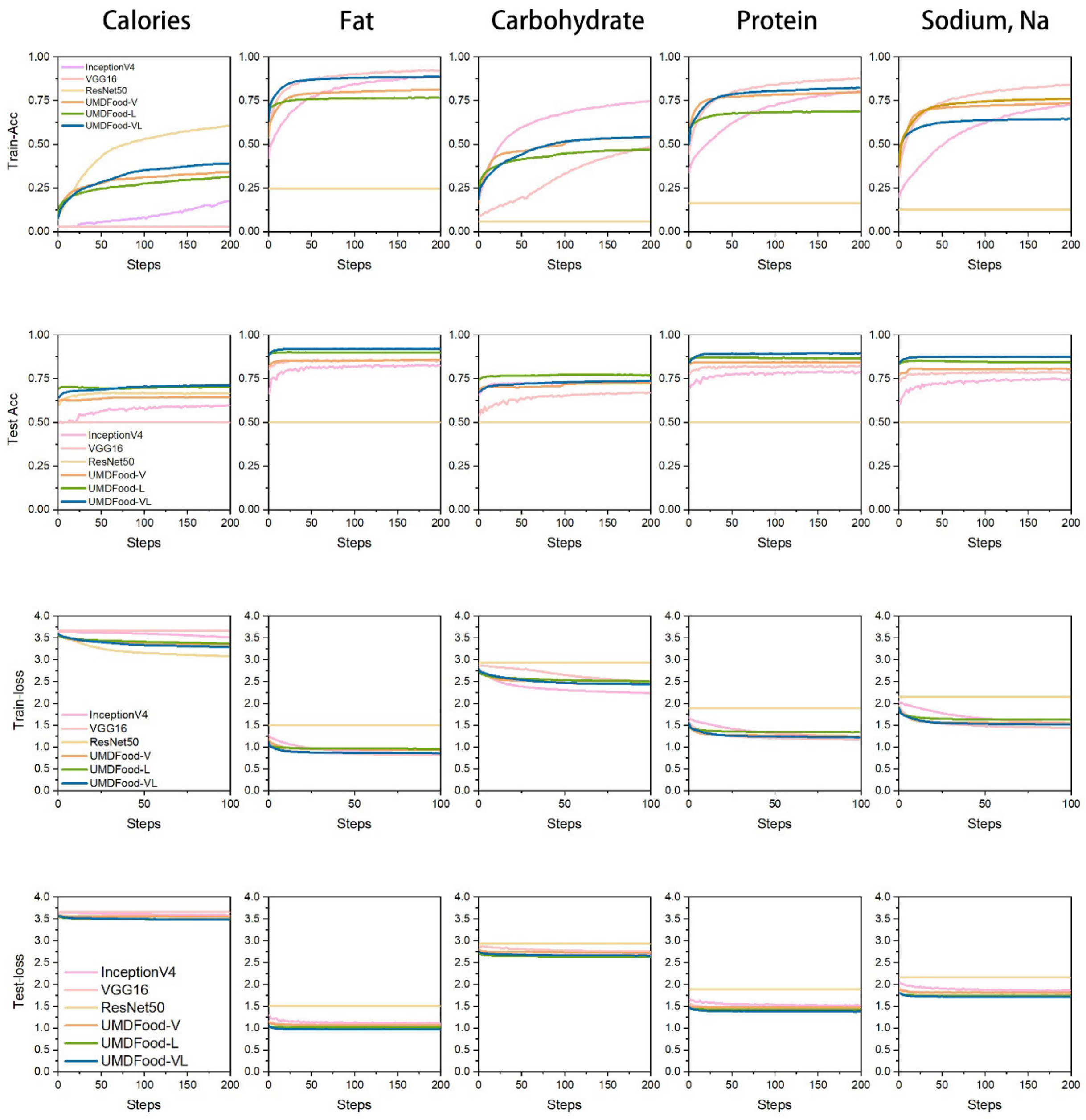


**Figure S6** Training accuracy, testing accuracy, training loss, and testing loss in the training process

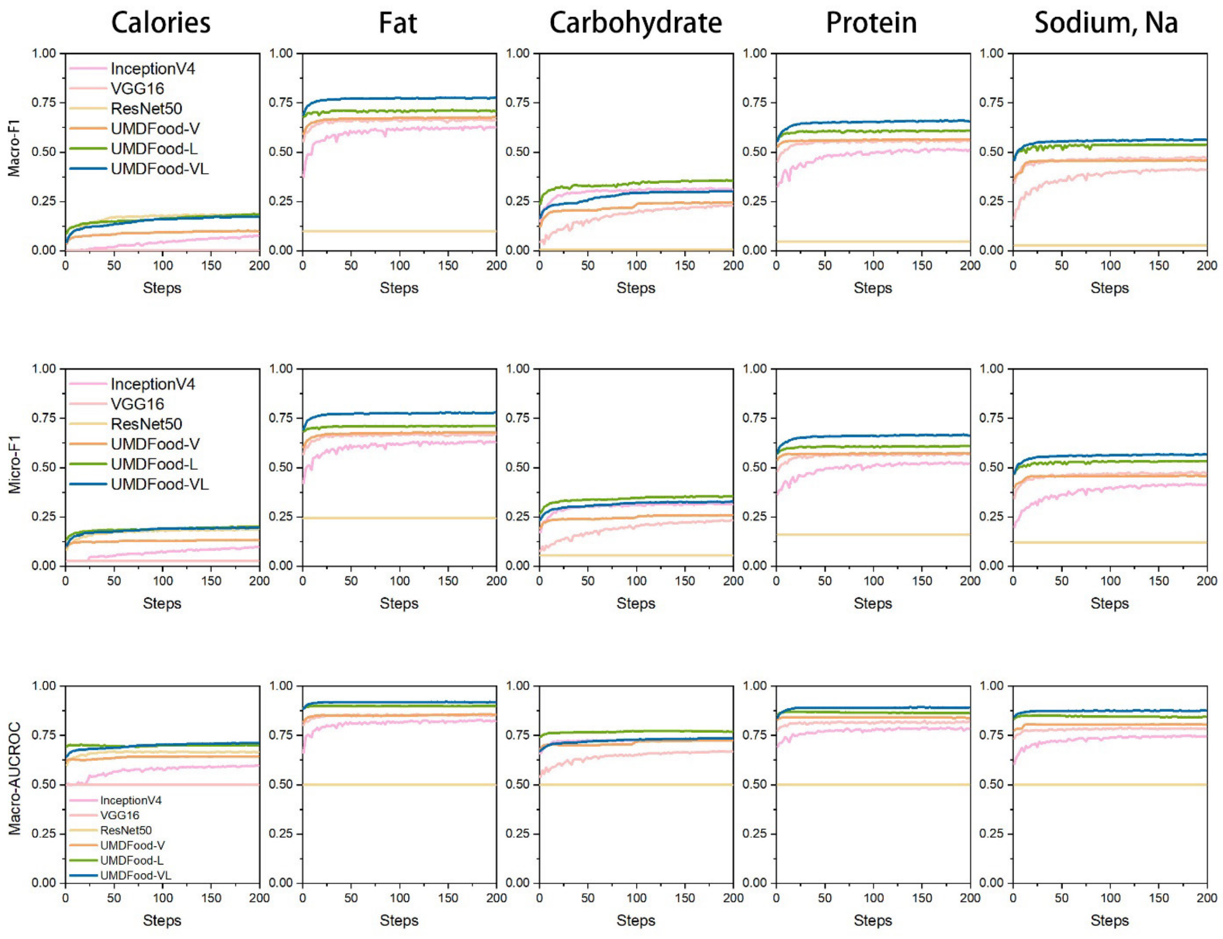


**Figure S7** Macro-F1, micro-F1 and Macro-AUCROC in the training process

In addition to the evaluation metrics reported in the main text, we have included the raw logs of the model training downloaded from the server. The batch size and number of epochs used for the models were set to 128 and 100, respectively. The UMDFood-VL model required a total training time of 35.90 hours for the five selected nutrients. The UMDFood-V model achieved the shortest training time, completing in 29.77 hours. Notably, all three UMDFood models had shorter training times compared to other deep learning models with similar computational power.

Furthermore, in our analysis, we reported additional metrics such as Macro-F1, Micro-F1, test loss, training accuracy, training loss, and others to provide a comprehensive evaluation of the models. These metrics offer further insights into the performance and training process of the models.

To calculate macro-F1, first calculate the F1 score for each class in the dataset (**Table S3**)(*1*). Then, calculate the average of the F1 scores for all classes. Unlike micro-F1, which weighs each class equally and calculates the overall F1 score, macro-F1 calculates the F1 score for each class and then takes the average. This means that macro-F1 gives equal weight to each class, regardless of how many instances there are for each class. Macro-F1 is a useful metric when the dataset is imbalanced, meaning that some classes have significantly fewer instances than others. In such cases, using micro-F1 can lead to misleading results as it tends to favor the performance of the majority class. By using macro-F1, the performance of the model is evaluated equally across all classes. Consistent with the AUCROC results reported in the main text, macro-F1 was significantly higher in the UMDFood-VL model than in the other models ($p<0.05$). The highest value was also for the fat content estimation task, reaching a maximum of 0.776. This signifies that the UMDFood-VL model is competent for imbalanced data.

Test loss and training loss were metrics used to evaluate the performance of a model during training and testing phases(*2*). Test loss refers to the error rate of the model during the testing phase. It is calculated by comparing the predicted output of the model to the actual output of a separate testing dataset that the model has not seen during training. The test loss provides an estimate of how well the model can generalize to new, unseen data. A low training loss and a low-test loss indicate that the model has learned to make accurate predictions both on the training and testing data, and is likely to perform well on new, unseen data as well. Training loss, on the other hand, refers to the error rate of the model during the training phase. It is calculated by comparing the predicted output of the model to the actual output of the training data. The goal during training is to minimize the training loss by adjusting the model's parameters using an optimization algorithm such as gradient descent. The training loss is typically used as the optimization objective during training. It is important to note that the goal of the training phase is to minimize the training loss, but this does not always guarantee good performance on the test data. If the model overfits to the training data, it may perform well on the training data but poorly on the test data, resulting in a high-test loss. Therefore, it is crucial to monitor both the training and test loss during training and make adjustments to the model as necessary to prevent overfitting. The combined results in Figure S6 show that the training process does not overfit.

To further analyze the advantages of multimodal data for nutrient estimation, here we counted 9 additional metrics, e.g., test accuracy, R2 score, Pearson coefficient(*3*), Spearman coefficient(*4*), Matthews coefficient(*5*), Kendall’s tau(*6*), micro Jaccard score, macro Jaccard score(*7*), and normalized discounted cumulative gain (NDCG)(*8*).

Test accuracy was a metric used to evaluate the performance of a classification model on a testing dataset. Test accuracy measures the percentage of correctly classified instances in the

testing dataset. To calculate test accuracy, the model's predicted outputs are compared to the actual outputs in the testing dataset. If the predicted output matches the actual output, the instance is considered correctly classified. The percentage of correctly classified instances out of the total number of instances in the testing dataset is the test accuracy. For example, here we have a testing dataset of 100 food products, and the model correctly classifies 85 of them in the right nutrition group. The test accuracy of the model would be 85%. Test accuracy is a useful metric for evaluating the overall performance of a classification model, especially when the classes in the dataset were balanced. However, when the dataset is imbalanced, test accuracy may not provide a complete picture of the model's performance.

R2 score, also known as the coefficient of determination, was a statistical metric used to evaluate the performance of a regression model. It takes values between 0 and 1, with a value of 1 indicating a perfect fit of the model to the data. R2 score was a widely used metric for evaluating regression models because it provides a simple measure of how well the model fits the data. Here we can clearly see that the cross-modal model shows significant superiority compared to single-image or single-component inputs. For example, there were no nutrients with unimodal inputs can have R2 values above 0.7, while cross-modal inputs with fat, protein and sodium have R2 above 0.7.

The Pearson correlation coefficient, also called the Pearson's r, is a statistical measure that quantifies the degree of linear association between two variables. It is denoted by the symbol "r" and ranges from -1 to 1. A value of -1 indicates a perfect negative correlation, where the two variables move in opposite directions. A value of 0 indicates no correlation between the variables, and a value of 1 indicates a perfect positive correlation, where the two variables move in the same direction. The Spearman's rank correlation coefficient, also called the Spearman's

rho, is a non-parametric measure of the strength and direction of the association between two variables. Unlike the Pearson correlation coefficient, which measures the linear relationship between two variables, the Spearman's rho measures the monotonic relationship, which is a generalization of the linear relationship. Here the results of Pearson and Spearman are highly similar. It is proved that the relationship between true and predicted values is highly linear, so the use of Pearson coefficient is more informative. In the same conclusion as other evaluation matrices, the UMDFood-VL model is significantly better than the unimodal model. The results for the prediction of fat, protein and sodium content reached 0.878, 0.883 and 0.872, respectively.

The Matthews correlation coefficient is a measure of the quality of binary (two-class) classifications that takes into account true positives (TP), true negatives (TN), false positives (FP), and false negatives (FN). The MCC ranges from -1 to +1, where a coefficient of +1 represents a perfect prediction, 0 no better than random prediction, and -1 indicates total disagreement between prediction and observation. Here we calculated the MCC by confusion matrix. In this approach, the confusion matrix is constructed by counting the number of instances that are correctly or incorrectly classified for each class. The MCC is then calculated by treating the confusion matrix as a two-class classification problem and applying the binary MCC formula.

Combining the results of other matrices, we can obtain that the cross-modal UMDFood-VL model was better to the unimodal model.  The reason may cause by 1. Better feature selection: The multimodal UMDFood-VL model may have selected a better set of features that were more informative for the classification task than unimodal models. 2. Better model architecture: The multimodal UMDFood-VL model may have a more complex or sophisticated architecture that is

better suited for the nutrients value estimation task than unimodal models. Kendall's tau, micro Jaccard score, macro Jaccard score, and NDCG result further support our conclusion.

**Table S3 Training log from server**

| Model | Nutrient | Created | Runtime | Batch size | Epochs | Macro AUCROC |
|---|---|---|---|---|---|---|
| UMDFood-VL | Calories | 2022-11-05T23:00:04.000Z | 5h 51m 45s | 128 | 100 | 0.709 |
| UMDFood-VL | Carbohydrates | 2022-11-06T08:46:17.000Z | 7h 11m 28s | 128 | 100 | 0.735 |
| UMDFood-VL | Fat | 2022-11-06T08:50:13.000Z | 9h 39m 18s | 128 | 100 | 0.921 |
| UMDFood-VL | Protein | 2022-11-06T02:06:00.000Z | 6h 27m 37s | 128 | 100 | 0.893 |
| UMDFood-VL | Sodium | 2022-11-06T02:19:07.000Z | 6h 43m 39s | 128 | 100 | 0.876 |
| UMDFood-V | Calories | 2022-11-06T11:01:02.000Z | 6h 49m 48s | 128 | 100 | 0.643 |
| UMDFood-V | Carbohydrates | 2022-11-06T22:08:22.000Z | 5h 13m 44s | 128 | 100 | 0.724 |
| UMDFood-V | Fat | 2022-11-07T07:52:48.000Z | 5h 15m 41s | 128 | 100 | 0.857 |
| UMDFood-V | Protein | 2022-11-06T15:05:56.000Z | 6h 20m 52s | 128 | 100 | 0.838 |
| UMDFood-V | Sodium | 2022-11-06T16:35:05.000Z | 6h 6m 25s | 128 | 100 | 0.805 |
| UMDFood-L | Calories | 2022-11-06T04:51:51.000Z | 6h 9m 9s | 128 | 100 | 0.701 |
| UMDFood-L | Carbohydrates | 2022-11-06T15:57:47.000Z | 6h 10m 33s | 128 | 100 | 0.769 |
| UMDFood-L | Fat | 2022-11-06T18:29:33.000Z | 7h 24m 7s | 128 | 100 | 0.898 |
| UMDFood-L | Protein | 2022-11-06T08:33:39.000Z | 6h 32m 15s | 128 | 100 | 0.864 |
| UMDFood-L | Sodium | 2022-11-06T09:02:48.000Z | 7h 32m 15s | 128 | 100 | 0.843 |
| ResNet50 | Calories | 2022-11-29T21:51:53.000Z | 8h 40m 14s | 128 | 100 | 0.667 |

| | | | | | | |
|---|---|---|---|---|---|---|
| ResNet50 | Carbohydrates | 2022-11-29T21:50:09.000Z | 7h 27m 0s | 128 | 100 | 0.726 |
| ResNet50 | Fat | 2022-11-30T17:10:03.000Z | 7h 17m 7s | 128 | 100 | 0.855 |
| ResNet50 | Protein | 2022-11-30T18:39:40.000Z | 7h 58m 33s | 128 | 100 | 0.82 |
| ResNet50 | Sodium | 2022-11-30T19:16:31.000Z | 8h 4m 27s | 128 | 100 | 0.783 |
| Inception_v4 | Calories | 2022-11-29T22:20:14.000Z | 9h 14m 49s | 128 | 100 | 0.596 |
| Inception_v4 | Carbohydrates | 2022-11-29T21:49:22.000Z | 8h 4m 8s | 128 | 100 | 0.669 |
| Inception_v4 | Fat | 2022-12-01T08:30:07.000Z | 8h 4m 11s | 128 | 100 | 0.823 |
| Inception_v4 | Protein | 2022-12-01T08:29:53.000Z | 7h 58m 34s | 128 | 100 | 0.783 |
| Inception_v4 | Sodium | 2022-12-01T08:11:54.000Z | 8h 4m 36s | 128 | 100 | 0.747 |
| VGG16 | Calories | 2022-11-29T21:54:30.000Z | 7h 54m 56s | 128 | 100 | 0.5 |
| VGG16 | Carbohydrates | 2022-11-29T19:37:57.000Z | 8h 59m 31s | 128 | 100 | 0.5 |
| VGG16 | Fat | 2022-11-30T09:23:31.000Z | 7h 46m 29s | 128 | 100 | 0.5 |
| VGG16 | Protein | 2022-11-30T09:36:12.000Z | 9h 3m 26s | 128 | 100 | 0.5 |
| VGG16 | Sodium | 2022-11-30T11:29:05.000Z | 7h 47m 23s | 128 | 100 | 0.5 |

**Table S4 Training metrics of different models**

| Model | Nutrient | Macro_F1 | Micro_F1 | Test loss | Training Acc | Training loss | Micro test AUCROC |
|---|---|---|---|---|---|---|---|
| UMDFood-VL | Calories | 0.172 | 0.195 | 3.48514655 | 0.389 | 3.290379349 | 0.712 |
| UMDFood-VL | Carbohydrates | 0.301 | 0.327 | 2.65179082 | 0.543 | 2.435530058 | 0.735 |
| UMDFood-VL | Fat | 0.776 | 0.779 | 0.9635588 | 0.887 | 0.856910402 | 0.92 |
| UMDFood-VL | Protein | 0.655 | 0.662 | 1.3792931 | 0.823 | 1.21994757 | 0.896 |
| UMDFood-VL | Sodium | 0.563 | 0.566 | 1.70588848 | 0.761 | 1.511267889 | 0.876 |
| UMDFood-V | Calories | 0.1 | 0.131 | 3.54765681 | 0.342 | 3.33647288 | 0.643 |
| UMDFood-V | Carbohydrates | 0.245 | 0.259 | 2.7184475 | 0.538 | 2.438789987 | 0.724 |
| UMDFood-V | Fat | 0.676 | 0.679 | 1.05982393 | 0.814 | 0.927633804 | 0.859 |
| UMDFood-V | Protein | 0.565 | 0.572 | 1.46719572 | 0.797 | 1.244157934 | 0.841 |
| UMDFood-V | Sodium | 0.459 | 0.459 | 1.80964814 | 0.735 | 1.535701279 | 0.805 |
| UMDFood-L | Calories | 0.184 | 0.201 | 3.47860969 | 0.312 | 3.367820826 | 0.701 |
| UMDFood-L | Carbohydrates | 0.355 | 0.354 | 2.62040433 | 0.469 | 2.505355175 | 0.77 |
| UMDFood-L | Fat | 0.707 | 0.711 | 1.01175995 | 0.766 | 0.95788263 | 0.9 |
| UMDFood-L | Protein | 0.609 | 0.609 | 1.41723999 | 0.687 | 1.340038933 | 0.867 |
| UMDFood-L | Sodium | 0.539 | 0.533 | 1.7315743 | 0.644 | 1.620742494 | 0.844 |
| ResNet50 | Calories | 0.182 | 0.187 | 3.49277506 | 0.604 | 3.078059561 | 0.667 |
| ResNet50 | Carbohydrates | 0.311 | 0.313 | 2.66636089 | 0.748 | 2.233596605 | 0.726 |

| | | | | | | | |
|---|---|---|---|---|---|---|---|
| ResNet50 | Fat | 0.661 | 0.668 | 1.0725377 | 0.923 | 0.819901217 | 0.857 |
| ResNet50 | Protein | 0.558 | 0.567 | 1.4730233 | 0.878 | 1.165645776 | 0.822 |
| ResNet50 | Sodium | 0.47 | 0.471 | 1.79944384 | 0.84 | 1.434225771 | 0.783 |
| Inception_v4 | Calories | 0.075 | 0.097 | 3.58247094 | 0.174 | 3.507091705 | 0.596 |
| Inception_v4 | Carbohydrates | 0.229 | 0.233 | 2.74605234 | 0.481 | 2.499395309 | 0.669 |
| Inception_v4 | Fat | 0.626 | 0.629 | 1.10944561 | 0.889 | 0.853566391 | 0.825 |
| Inception_v4 | Protein | 0.511 | 0.523 | 1.5172292 | 0.804 | 1.238911009 | 0.786 |
| Inception_v4 | Sodium | 0.409 | 0.414 | 1.85632102 | 0.727 | 1.546142354 | 0.747 |
| VGG16 | Calories | 0.001 | 0.027 | 3.65482417 | 0.026 | 3.655799853 | 0.5 |
| VGG16 | Carbohydrates | 0.006 | 0.055 | 2.92697654 | 0.056 | 2.925788154 | 0.5 |
| VGG16 | Fat | 0.098 | 0.242 | 1.50131614 | 0.244 | 1.499634765 | 0.5 |
| VGG16 | Protein | 0.046 | 0.161 | 1.88302979 | 0.162 | 1.881920538 | 0.5 |
| VGG16 | Sodium | 0.027 | 0.121 | 2.15254955 | 0.125 | 2.148902617 | 0.5 |

**Table S5 Supporting training metrics of different UMDFood model (part 1)**

| Model | Nutrient | Test acc | R2_score | Pearson coef | Spearman coef | Matthews coef |
|---|---|---|---|---|---|---|
| UMDFood-VL | Calories | 0.195 | 0.282 | 0.669 | 0.671 | 0.174 |
| UMDFood-VL | Carbohydrates | 0.327 | 0.514 | 0.760 | 0.749 | 0.289 |
| UMDFood-VL | Fat | 0.779 | 0.754 | 0.876 | 0.878 | 0.705 |
| UMDFood-VL | Protein | 0.662 | 0.766 | 0.881 | 0.883 | 0.595 |
| UMDFood-VL | Sodium | 0.566 | 0.743 | 0.873 | 0.872 | 0.504 |
| UMDFood-V | Calories | 0.201 | 0.032 | 0.636 | 0.609 | 0.184 |
| UMDFood-V | Carbohydrates | 0.354 | 0.343 | 0.731 | 0.710 | 0.325 |
| UMDFood-V | Fat | 0.711 | 0.488 | 0.795 | 0.781 | 0.631 |
| UMDFood-V | Protein | 0.609 | 0.668 | 0.826 | 0.827 | 0.546 |
| UMDFood-V | Sodium | 0.533 | 0.621 | 0.827 | 0.816 | 0.476 |
| UMDFood-L | Calories | 0.131 | -0.051 | 0.501 | 0.499 | 0.109 |
| UMDFood-L | Carbohydrates | 0.259 | 0.047 | 0.541 | 0.544 | 0.216 |
| UMDFood-L | Fat | 0.679 | 0.512 | 0.755 | 0.755 | 0.572 |
| UMDFood-L | Protein | 0.571 | 0.565 | 0.780 | 0.782 | 0.485 |
| UMDFood-L | Sodium | 0.459 | 0.424 | 0.713 | 0.713 | 0.382 |

**Table S6 Supporting training metrics of different model (part 2)**

| Model | Nutrient | Kendall's tau | Weighted Jaccard score | Macro Jaccard score | NDCG |
|---|---|---|---|---|---|
| UMDFood-VL | Calories | 0.531 | 0.106 | 0.106 | 0.444 |
| UMDFood-VL | Carbohydrates | 0.622 | 0.193 | 0.192 | 0.581 |
| UMDFood-VL | Fat | 0.825 | 0.651 | 0.645 | 0.907 |
| UMDFood-VL | Protein | 0.806 | 0.515 | 0.504 | 0.844 |
| UMDFood-VL | Sodium | 0.777 | 0.403 | 0.401 | 0.792 |
| UMDFood-V | Calories | 0.488 | 0.113 | 0.113 | 0.453 |
| UMDFood-V | Carbohydrates | 0.594 | 0.235 | 0.234 | 0.605 |
| UMDFood-V | Fat | 0.722 | 0.573 | 0.565 | 0.876 |
| UMDFood-V | Protein | 0.748 | 0.470 | 0.459 | 0.815 |
| UMDFood-V | Sodium | 0.719 | 0.382 | 0.380 | 0.768 |
| UMDFood-L | Calories | 0.380 | 0.057 | 0.057 | 0.382 |
| UMDFood-L | Carbohydrates | 0.438 | 0.147 | 0.147 | 0.537 |
| UMDFood-L | Fat | 0.691 | 0.524 | 0.519 | 0.856 |
| UMDFood-L | Protein | 0.691 | 0.415 | 0.407 | 0.798 |
| UMDFood-L | Sodium | 0.611 | 0.303 | 0.302 | 0.72 |

## Section 4 Comparison between different food categories among UMDFood-90k

We calculated the accuracy of the different models in different food categories (**Fig. S8-S12**). Firstly, for the calorie content prediction results, all models were the most accurate for water. This is due to the fact that water has a calorie content of 0 and a single ingredient. The worst prediction of the model is VGG16 model on bread & buns, as low as 0.256, and the best prediction of UMDFood-V, as higher as 0.873. The comparison of different models shows that the VGG16 model has the worst prediction results in all categories, while the UMDFood-L has the best results. Of the 33 subcategories counted, 13 were the best. UMDFood-VL followed closely with 11 of the best. This result indicated that the estimation of the calorie value was mainly based on the ingredient list of the food, and the information provided by the front-of-package image may instead be a distraction from the estimation(*9*). Protein and sodium content estimation results were highly consistent with calorie estimation results.

As mentioned in manuscript, fat estimation result is the best among reported nutrients. This is due to the fact that many species have zero fat content, thus greatly reducing the difficulty of model learning. For example, canned fruits, sodas and water, all models achieved accuracy close to 100%. In sharp contrast, to some challenge categories (i.e., cookies & biscuits). UMDFood models showed significant improvement when compared to other models, e.g., UMDFood-VL increased 99% when compared to ResNet50 and InceptiveV4 model.

In the estimation results of carbohydrate content estimation, UMDFood-L showed advantages in more groups. In the estimation of carbohydrate content, UMDFood-L obtained the best estimation results in all 15 food groups and the advantage was more than 10% in 4 groups. This

result suggests that the model's estimation of carbohydrate content is largely derived from the composition, while the image information is more used as noise for model training. However, it is also worth noting that none of the model's estimation results for carbohydrates were satisfactory, and only two food categories had less than 20% estimation error in the proportion of samples less than 0.1, i.e., Baking Decorations & Dessert Toppings.

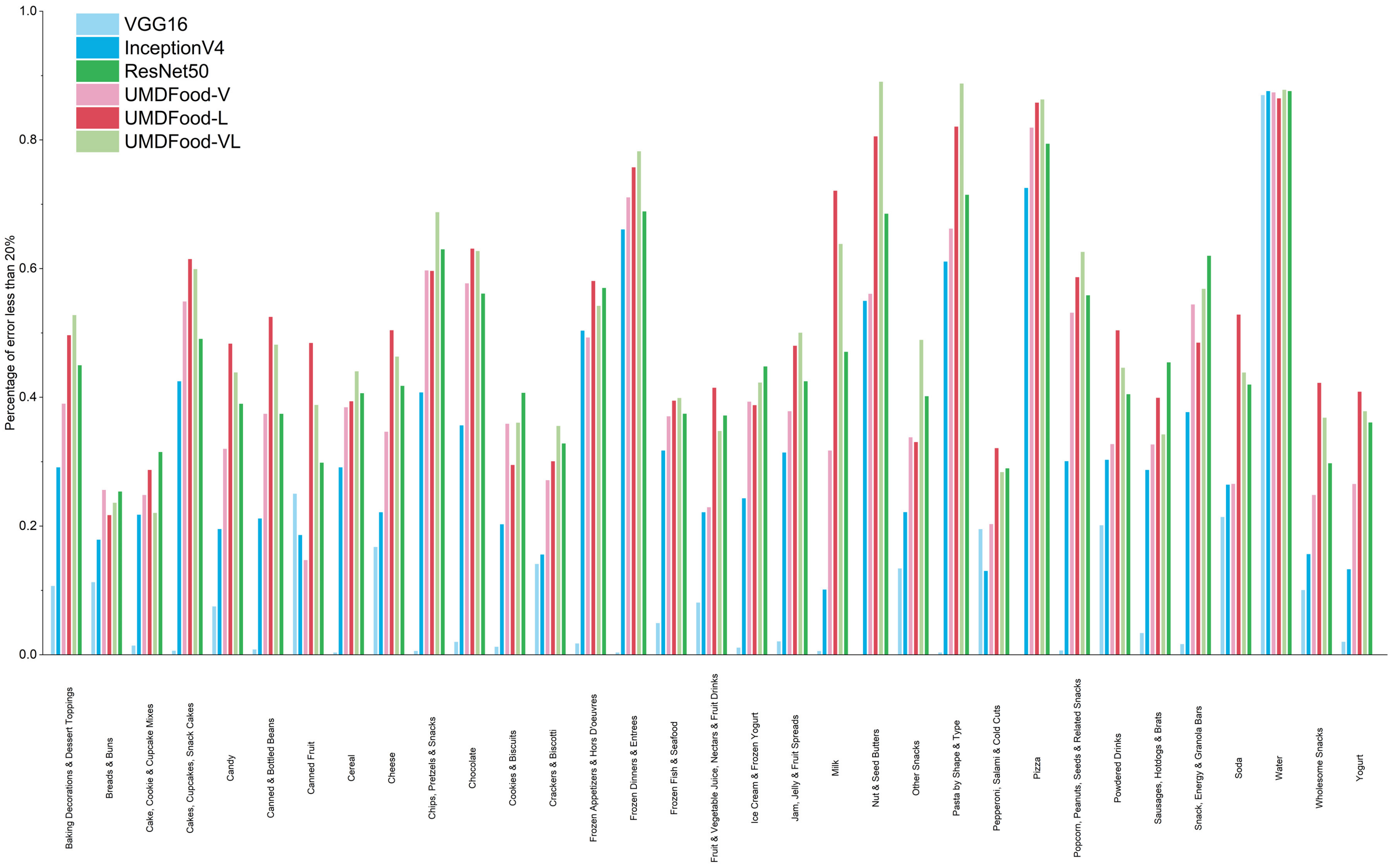


**Figure S8** Calories estimation results of different categories in UMDFood-90k

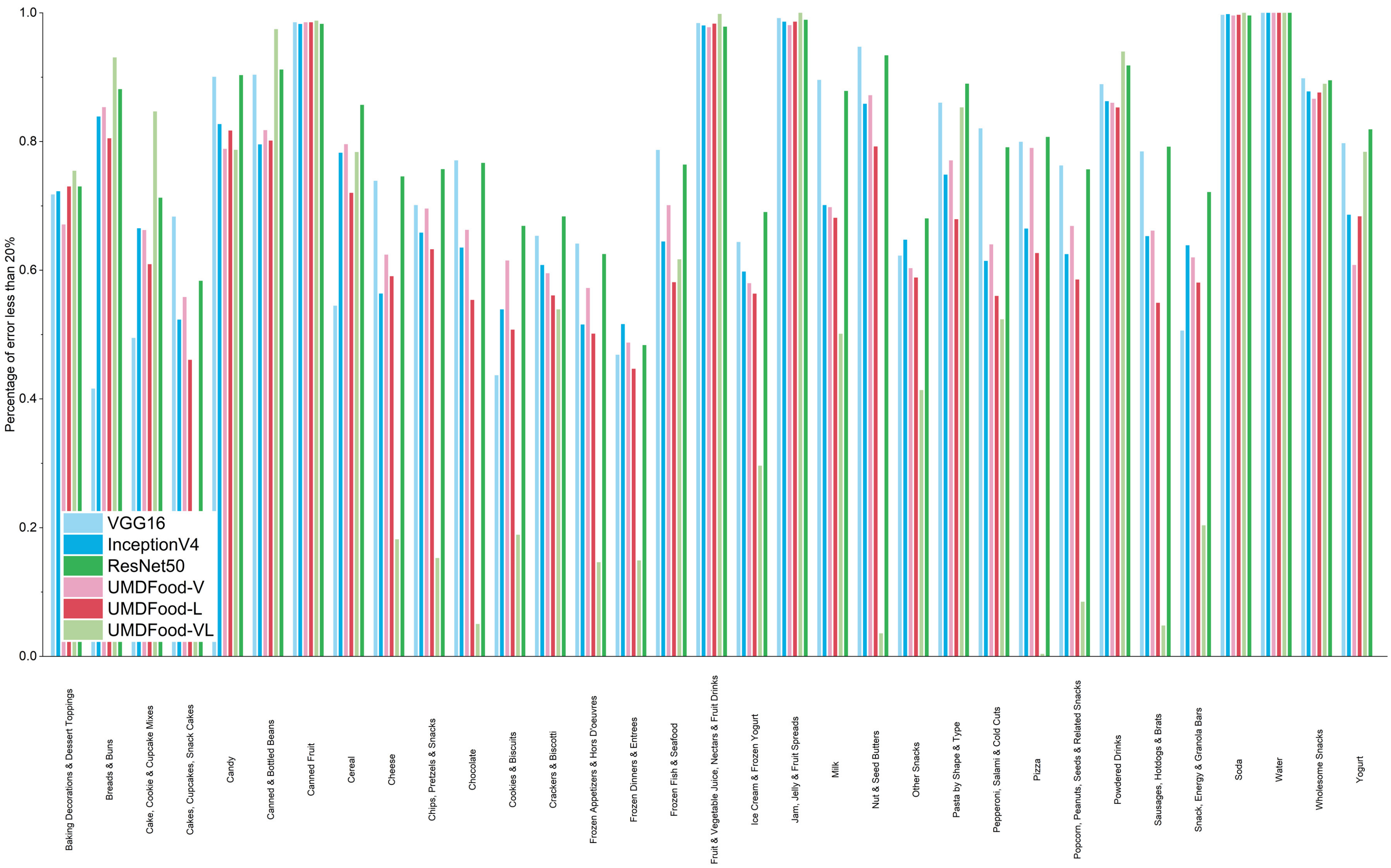


**Figure S9** Fat content estimation results of different categories in UMDFood-90k

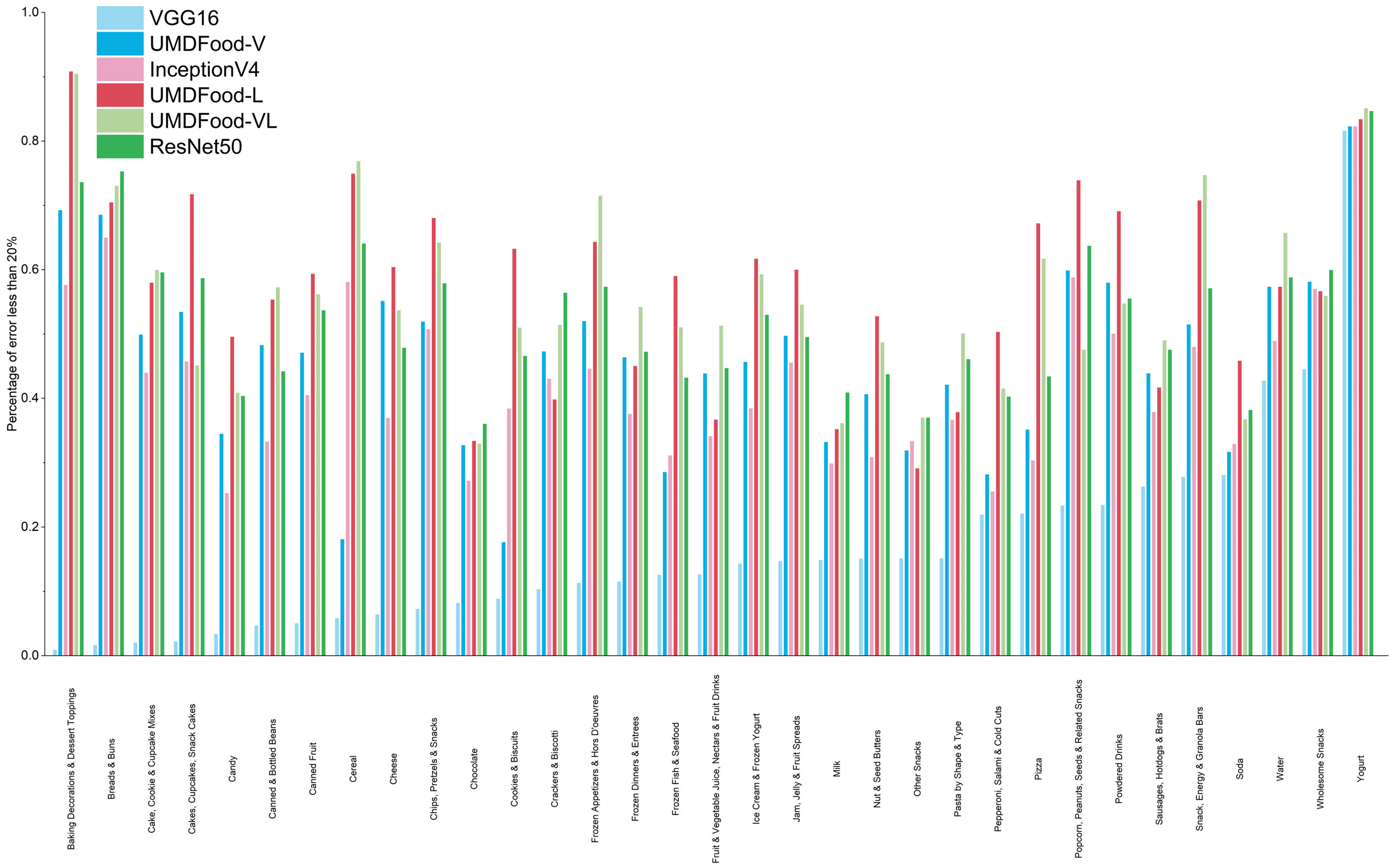


**Figure S10** Carbohydrate content estimation results of different categories in UMDFood-90k

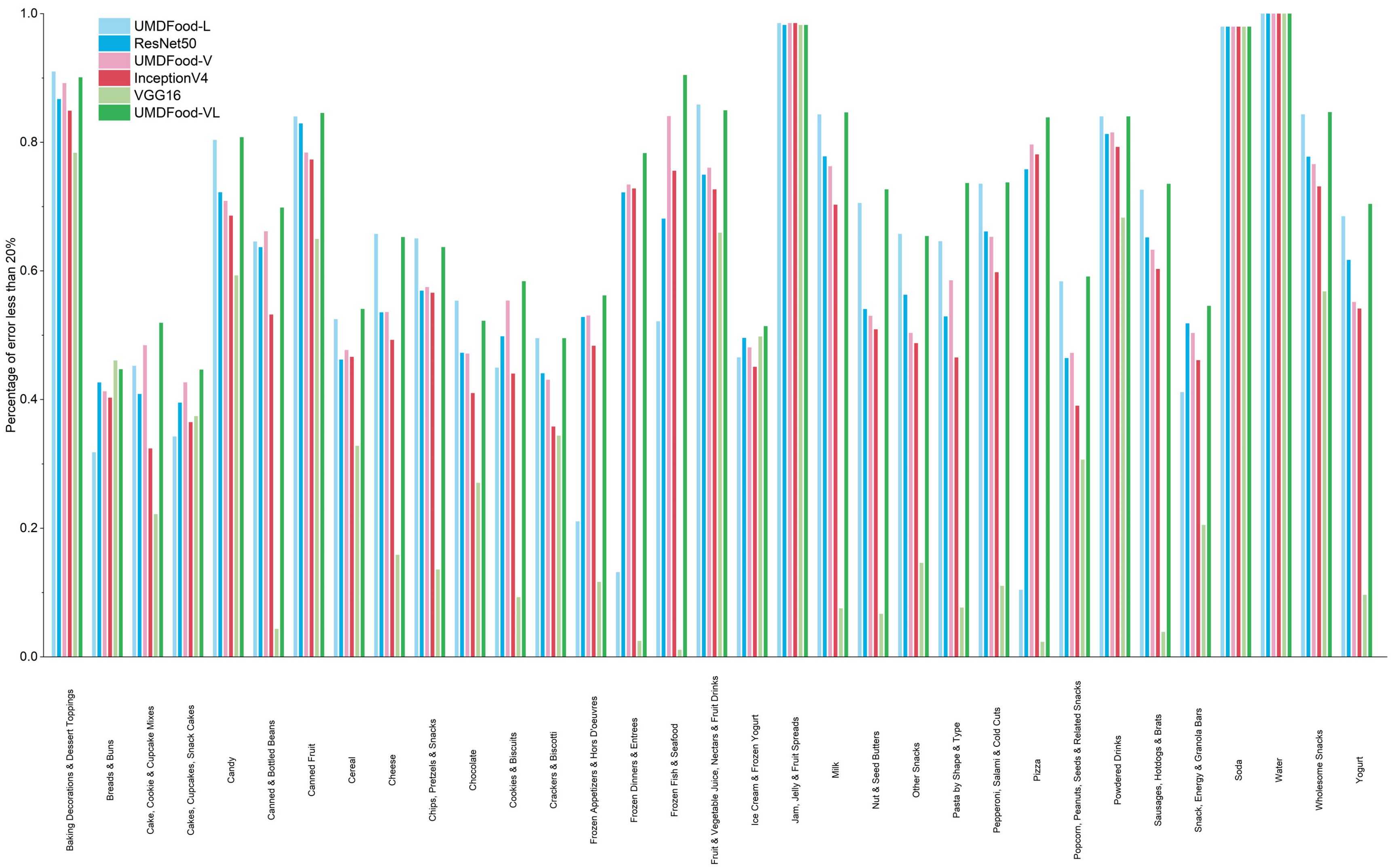


**Figure S11** Protein content estimation results of different categories in UMDFood-90k

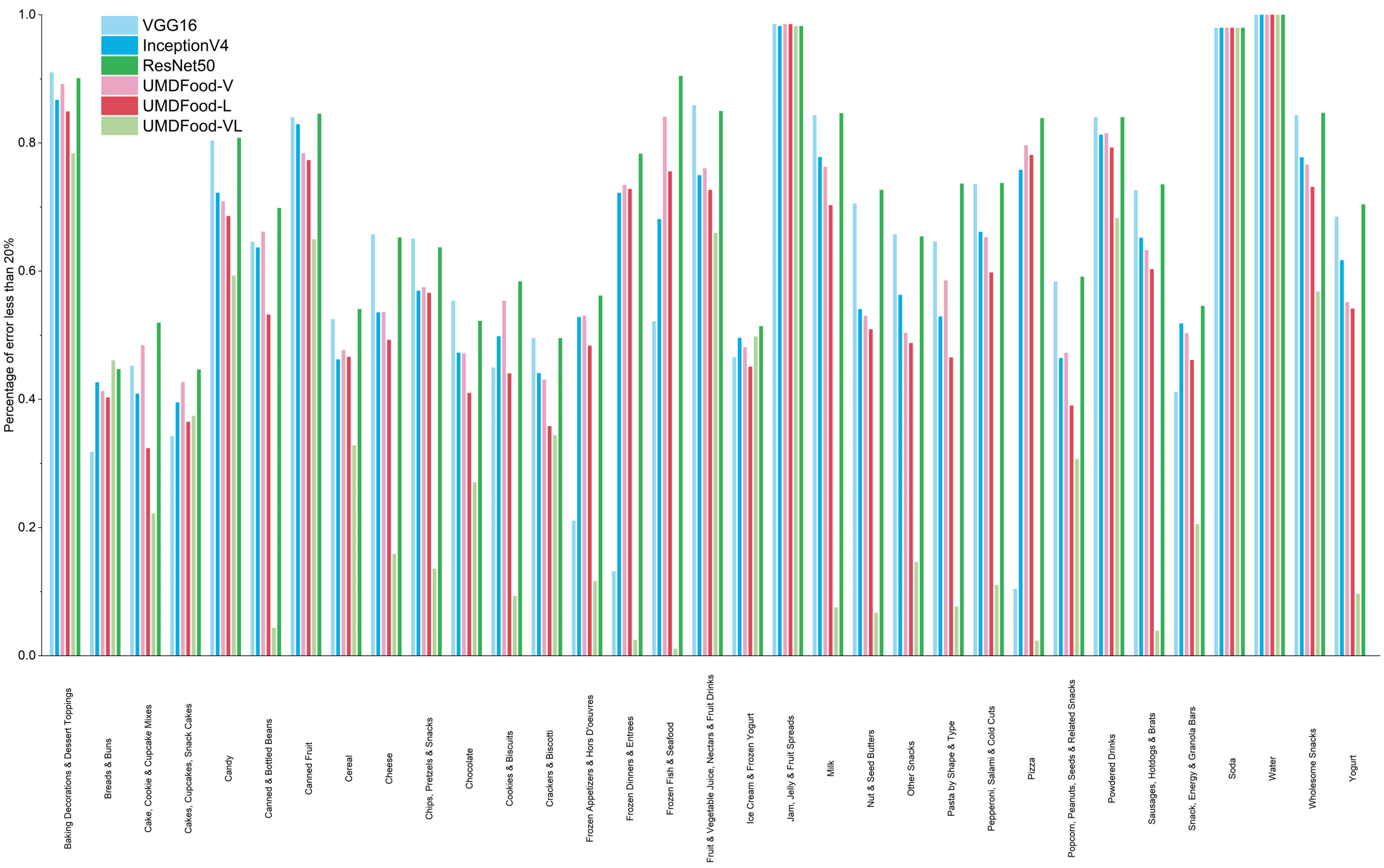


**Figure S12** Sodium content estimation results of different categories in UMDFood-90k

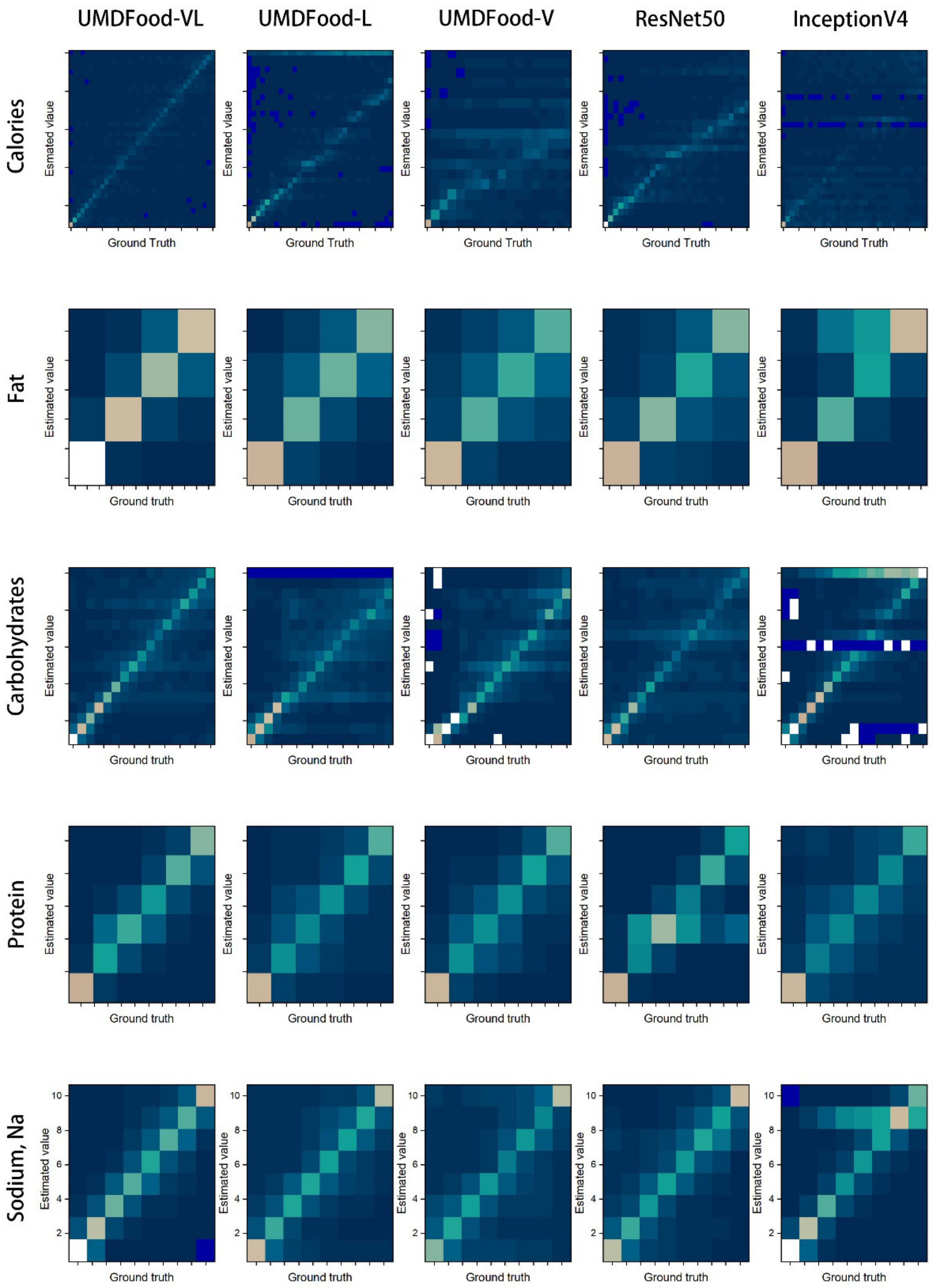


**Figure S13** Confusion matrix of different models for different nutrients

## Section 5 Chemical analysis result of beverage products

In the comparison of the chemical analysis values of the four nutrients and the two data values, we excluded the fat content from the analysis as it was highly concentrated, and most beverage fat contents were 0. We used a 3D scatter plot to visualize the relationships, where the radius of the scatter represents the standard deviation of the chemical analysis results. A more linear scatter distribution indicates a higher correlation between the three relationships.

The calorie content distribution appeared to be relatively evenly distributed. Values with larger deviations tended to have higher chemical analysis values compared to the model predicted values and BFPD values. This suggests that producers may underestimate the calorie content when labeling their products (**fig. S14a**).

The distribution of carbohydrate content differed significantly from that of calories. The majority of beverage products had carbohydrate distributions concentrated in the range of 10g/100mL to 15g/100mL, resulting in no significant difference between the BFPD values, UMDFood-VL values, and the chemical analysis values (**fig. S14b**).

The distribution of protein content was correlated with the content. The figure only includes 15 samples due to the presence of 35 beverage samples with a protein content of 0. Samples with high protein content exhibited larger deviations between the three values. Conversely, for samples with low protein content (less than 5g/100mL), the correlation between the three values was stronger (**fig. S14c**).

Regarding the estimation of sodium content, the sample distribution was correlated with the content. There were more samples with low sodium content and fewer samples with high sodium content. The error in sodium content testing was higher due to the assay used. For samples with

sodium content above 40mg/100mL, the correlation between the three values was poor. Additionally, there was a significant error between the chemical analysis values and the BFPD values used for training. To improve accuracy, it may be beneficial in subsequent optimization to directly test data for products with high sodium content and provide that information to the machine for learning (**fig. S14d**).

These observations highlight potential areas for improvement in future iterations of the model and suggest the need for better data collection and testing methods, particularly for nutrients with specific distribution patterns or high variability in the samples.

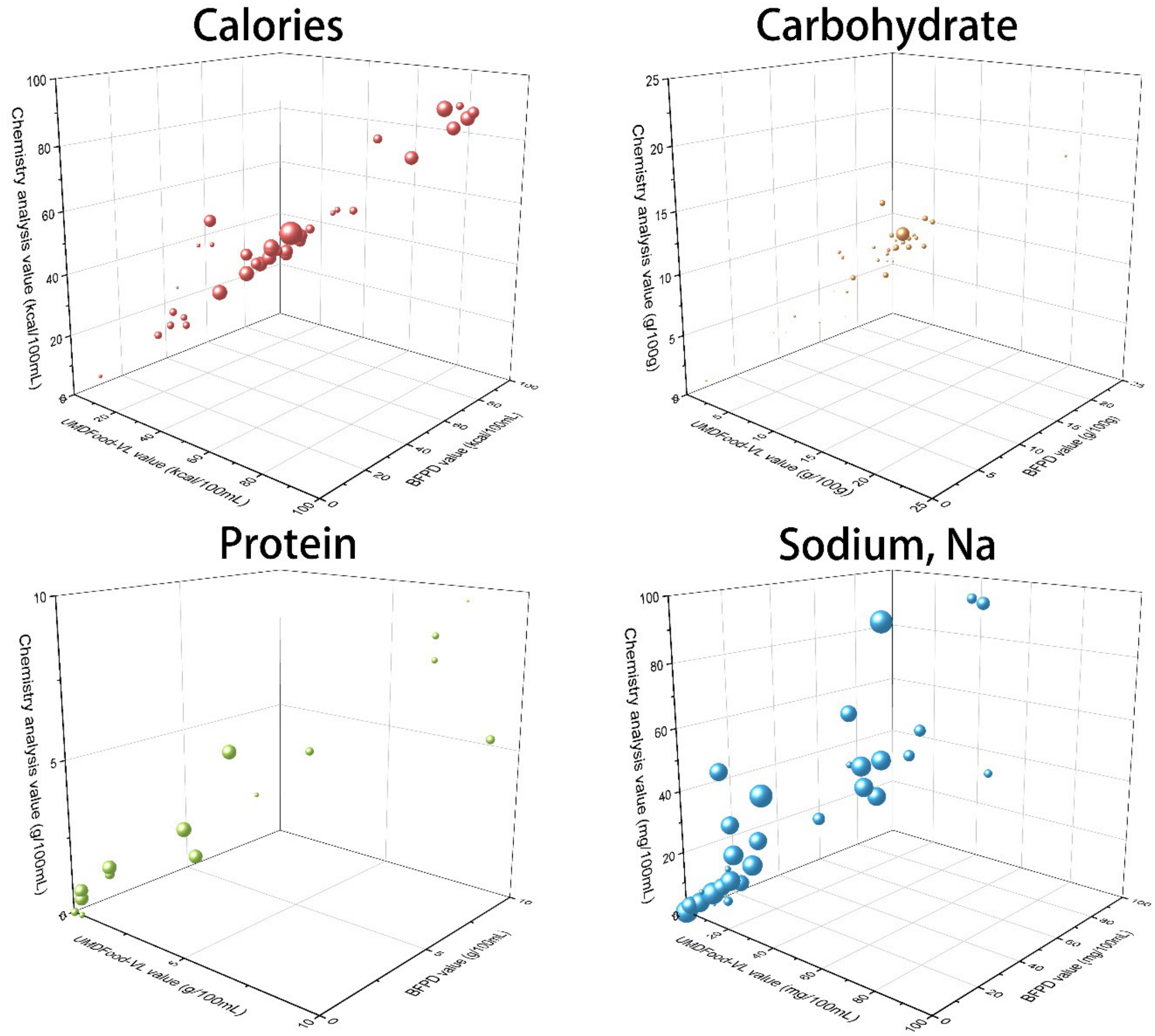


**Figure S14** Comparison of nutrient value between USDA-BFPD, UMDFood-VL and chemical analysis value. a, calories, b, carbohydrate by different, c, protein, d, sodium.

## References:


1. D. Chicco, G. Jurman, The advantages of the Matthews correlation coefficient (MCC) over F1 score and accuracy in binary classification evaluation. *BMC genomics* 21, 1-13 (2020).
2. K. Janocha, W. M. Czarnecki, On loss functions for deep neural networks in classification. *arXiv preprint arXiv:1702.05659*, (2017).
3. J. Benesty, J. Chen, Y. Huang, I. Cohen, "Pearson correlation coefficient" in *Noise reduction in speech processing* (Springer, 2009), pp. 1-4.
4. M. A. Skinnider, R. G. Stacey, D. S. Wishart, L. J. Foster, Chemical language models enable navigation in sparsely populated chemical space. *Nature Machine Intelligence* 3, 759-770 (2021).
5. N. Amin, A. McGrath, Y.-P. P. Chen, Evaluation of deep learning in non-coding RNA classification. *Nature Machine Intelligence* 1, 246-256 (2019).
6. T. Kieu, B. Yang, C. S. Jensen, in *2018 19th IEEE international conference on mobile data management (MDM)*. (IEEE, 2018), pp. 125-134.
7. S. Dabiri, K. Popuri, C. Ma, V. Chow, E. M. C. Feliciano, B. J. Caan, V. E. Baracos, M. F. Beg, Deep learning method for localization and segmentation of abdominal CT. *Computerized Medical Imaging and Graphics* 85, 101776 (2020).
8. O. Baker, Q. Yuan, in *2021 IEEE International Conference on Computing (ICOCO)*. (IEEE, 2021), pp. 31-36.
9. P. Ma, A. Li, N. Yu, Y. Li, R. Bahadur, Q. Wang, J. K. Ahuja, Application of machine learning for estimating label nutrients using USDA Global Branded Food Products Database,(BFPD). *Journal of Food Composition and Analysis*, 103857 (2021).
